# Dachsous cadherin related 1 (DCHS1) is a novel biomarker for immune infiltration and epithelial-mesenchymal transition in endometrial cancer via pan-cancer analysis

**DOI:** 10.1186/s13048-024-01478-1

**Published:** 2024-08-09

**Authors:** Cai Meijuan, Min Fang, Wang qian

**Affiliations:** 1 Department of Clinical Laboratory, Qilu Hospital of Shandong University, Jinan, Shandong China; 2 Department of Clinical Laboratory, Qilu Hospital of Shandong University (Qingdao), No.758 Hefei Road, Qingdao, 266035 Shandong China; 3 Department of Gynecology, Qingdao Women’s and Children’s Hospital, Qingdao University, Qingdao, Shandong China

**Keywords:** DCHS1, Immune infiltration, Biomarker, EMT, UCEC

## Abstract

**Background:**

Dachsous cadherin related 1 (DCHS1) is one of calcium-dependent adhesion membrane proteins and is mainly involved in the development of mammalian tissues. There is a lack of more detailed research on the biological function of DCHS1 in pan-cancer.

**Materials and methods:**

We evaluated the expression, the prognostic value, the diagnostic value and genomic alterations of DCHS1 by using the databases, including TCGA, UALCAN, HPA, GEPIA2.0 and GSCA. We employed the databases of UCSC, TIMER2.0, TISIDB, GSCA to analyze the association between DCHS1 expression and the immune microenvironment, stemness, TMB, MSI and anticancer drug sensitivity. BioGRID, STRING and GEPIA2.0 were used to perform protein interaction and functional enrichment analysis. Real-time quantitative PCR, CCK8, Transwell assay and Western blot were performed to determine the function of DCHS1 in UCEC.

**Results:**

DCHS1 is differentially expressed in many cancers and its expression is significantly associated with tumor prognosis and diagnosis. DCHS1 expression was significantly correlated with the infiltration of cancer-associated fibroblasts (CAFs), Endothelial cell (ECs), and Hematopoietic stem cell in most cancers. In addition, DCHS1 was significantly associated with sensitivity to many antitumor drugs. Functional enrichment analysis revealed that DCHS1-related proteins were involved in Focal adhesion, Endometrial cancer and Wnt signaling pathway. GSEA results showed that DCHS1 was related to epithelial-mesenchymal transition (EMT) in many cancers. In vitro experiments in UCEC showed that DCHS1 regulated cell proliferation, migration and EMT.

**Conclusions:**

Our findings indicated that DCHS1 might be a novel prognostic and diagnostic biomarker and immunotherapy target, and plays an important role in the proliferation, migration and EMT in UCEC.

**Supplementary Information:**

The online version contains supplementary material available at 10.1186/s13048-024-01478-1.

## Introduction

Human cancers are widely acknowledged as a global problem because of its high prevalence and morbidity rates [1]. Although various innovative treatments of cancer have made great outcome, such as chemotherapy, radiation, immunotherapy, and targeted therapies, the prognosis and survival rate individuals in advanced stages remains dismal [2]. Increasing studies have reported the common underlying mechanisms of tumor progression and identified many biomarkers for prognosis and diagnosis in various cancers [3–5]. However, single cancer-targeting studies limit our understanding of the underlying mechanisms of tumorigenesis. Thus, identifying the valuable pan-cancer genes would be crucial to reveal the mechanism for development and occurrence in malignant cancers [6, 7], and provide new insight for cancer therapy.

Cadherins are one of the most important adhesion molecules mediating cell-cell junctions and form a superfamily of 114 calcium-dependent adhesion membrane proteins [8]. Dachsous originally discovered in *Drosophila*, is one of the largest members of the cadherin superfamily [9]. Boundary of Dachsous activity induces cell proliferation by modulating the Hippo signaling pathway in *Drosophila* [10]. In vertebrate, there were two Dachsous proteins (DCHS1-2). DCHS1 encodes a calcium-dependent cell-cell adhesion protein with a single peptide, 27 cadherin repeat domains and a unique cytoplasmic region [11]. Previous study revealed that DCHS1 is mainly involved in the development of mammalian tissues and the preservation of stem cell progenitor pools. DCHS1 regulates cell movements such as convergence-extension and cell migration in planar cell polarity [12], as well as controls tissue proliferation and stem cell activity by the regulation of YAP/TAZ independently of the Hippo kinase cascade [13]. In gut development, FAT4-DCHS1 signaling axis is required for proper vilification and mesenchymal clustering [14]. DCHS1 together with bone morphogenetic proteins, fibroblast growth factors and retinoic acid is involved in external ear development via Wnt (wingless/INT) signaling pathway [15]. Based on the whole-exome sequencing of papillary thyriod microcarcinomas, researchers found that nonsynonymous mutations of 13 cell adhesion-related genes, including DCHS1, were only observed in the aggressive group [16], but the function of DCHS1 in pan-cancer was limited. Therefore, we conducted the expression, the prognostic, the diagnostic and epigenetic changes of DCHS1 in pan-cancer. In addition, we assessed the relevance of DCHS1 expression to immune signature and immune-regulated genes. Moreover, the association between DCHS1 expression and tumor mutation burden (TMB), microsatellite instability (MSI), stemness and drug sensitivity was examined to explore its value on tumor immunotherapy. We also performed GSEA pathway enrichment analysis and found that DCHS1 is associated with the EMT in various cancers. Then we conducted some experiments to verify the function of DCHS1 in endometrial cancer in vitro.

## Materials and methods

### DCHS1 expression analysis

We collected the transcriptome data of DCHS1 from pan-cancer samples in TCGA by using the UCSC Xena (https://xena.ucsc.edu). DCHS1 mRNA expression levels in cancers and normal tissues were observed by the ggplot2 (v3.3.6) and stats (v4.2.1) R packages. The expression levels were displayed by using a log2(TPM + 1) scale. The protein expression of DCHS1 in pan-cancer was investigated by the clinical proteomic tumor analysis consortium (CPTAC) in UALCAN database (http://ualcan.path.uab.edu/analysis.html) [17]. The Human Protein Atlas (HPA) (https://www.proteinatlas.org/) was employed to examine the intensity of DCHS1 immunohistochemical staining in several cancer tissues, including breast carcinoma (BRCA), prostate adenocarcinoma (PRAD), liver hepatocellular carcinoma (LIHC) and uterine corpus endometrial carcinoma (UCEC) [18].

### Prognostic and diagnostic assessment in pan-cancer

GEPIA 2.0 (http://gepia2.cancer-pku.cn/#general) was used to analyze the prognostic value of DCHS1 expression, including overall survival (OS) and disease free survival (RFS) [19]. TCGA tumor patients were divided into two groups (high-expression and low-expression) based on the cut-off value. The best cut-off value was identified by using the function “surv_cutpoint” in R package “survminer (v0.4.9)”. The hazards ratio was calculated based on Cox proportional hazards Model. A heatmap showing the survival analysis results across pan-cancers was obtained from the “Survival Map” module in “Survival analysis”. The receiver operating characteristic (ROC) analysis was used to compare the prediction accuracy of DCHS1 analyzed by the R packages “pROC” and “ggplot2”. In principles, AUC values above 0.8 are considered to be of high reliability.

### Genomic alteration analysis

Gene Set Cancer Analysis (GSCA) (http://bioinfo.life.hust.edu.cn/GSCA/#/) is an integrated platform for genomic, pharmacogenomic, and immunogenomic gene set cancer analysis [20]. GSCA database was used to analyze single nucleotide variation (SNV), copy number variation (CNV), methylation and drug sensitivity of DCHS1 in pan-cancer and the correlations with gene expression. The cBioportal (https://www.cbioportal.org/) was used for genetic alteration analysis in UCEC [21]. The mutation types and frequency were obtained from the “mutations” module. The effect of genetic alterations on survival was obtained from “Comparison/Survival” module.

### Immune infiltration analysis

We used “Cancer Exploration” module in TIMER 2.0 (http://timer.cistrome.org/) to examine the different expression profiles of DCHS1 between tumor and adjacent normal tissues across all TCGA cohorts [22]. “Immune Association” module was employed to evaluate the correlation between DCHS1 expression and immune infiltration based on several immune deconvolution algorithms, such as TIMER, EPIC, MCPCOUNTER, XCELL and TIDE. TISIDB (http://cis.hku.hk/TISIDB/) was used to analyze the correlation between DCHS1 and immune subtypes [23]. The associations between molecular profile and immune signature, tumor immune infiltration, stemness, tumor mutation burden (TMB) and microsatellite instability (MSI) were estimated by using UCSCXenaShiny (https://shixiangwang.shinyapps.io/ucscxenashiny/).

### Gene enrichment analysis

BioGRID (https://thebiogrid.org/) is a biomedical interaction repository with data compiled through comprehensive curation efforts [24]. We obtained 15 DCHS1-interacted proteins by using BioGRID. We used STRING to get 50 DCHS1-interacted proteins by setting “max number of interactors to show: no more than 50 interactors”. GEPIA 2.0 was used to obtain the top 100 DCHS1-correlated genes in “similar genes detection” module. Gene ontology (GO) and Kyoto encyclopedia of genes and genome (KEGG) enrichment analysis were performed by “clusterProfiler (v4.4.4)”, “ggplot2(v3.3.6)”, “igraph(v1.3.4)” and “ggraph(v2.1.0)” R-packages. Gene Set Enrichment Analysis (GSEA) analysis was performed by using the R-package “clusterProfiler (v3.14.3)”. False discovery rate (FDR) < 0.25 and p.adjust < 0.05 was considered as significantly enriched.

### Specimen collection

A total of eight pairs of EC and adjacent tissues were collected from patients who underwent surgical resection at Qilu Hospital of Shandong University (Qingdao) from December 2021 to March 2023. None of patients received hormone therapy, intrauterine devices, chemotherapy or radiotherapy for at least 6 months prior to surgery. All samples were evaluated by at least two pathologists according to World Health Organization guidelines. This work was approved by the Ethics Committees of Qilu Hospital of Shandong University (Qingdao) (Approval No. KYLL-2021027). Permission for the use of tissues was obtained from all patients prior to the surgery.

### Cell culture and transfection

The human normal endometrial hEM15A stromal cell (No. YS2459C) were purchased from YaJi Biological (Shanghai, China). RL95-2 (ZQ0362), Ishikawa (ISK, ZQ0472), HEC-1B (ZQ0364), AN3CA (ZQ0471) and KLE (ZQ0473) cells were purchased from Shanghai Zhong Qiao Xin Zhou Biotechnology Co., Ltd (Shanghai, China). hEM15A, RL95-2 and KLE cells were incubated in DMEM containing 10% FBS. ISK cells were cultured in RPMI-1640 medium supplement with 10% FBS. HEC-1B and AN3CA cells were incubated in MEM containing 10% FBS. HEC-1A (BNCC338711) cells were purchased from BeNa Culture Collection (Beijing, China) and cultured in McCoy’s 5a medium containing 10% FBS. For DCHS1 silencing, ISK or AN3CA cells were transfected with different siRNA. Negative control scrambled siRNA (NC siRNA) and DCHS1 siRNA (siRNA#1, siRNA#2 and siRNA#3) were synthesized by Sangon Biotech (Shanghai, China). The siRNA sequences were as follows: NC siRNA sense: 5’-uucuccgaacgugucacgutt-3’, antisense: 5’-acgugacacguucggagaatt-3’; DCHS1siRNA#1 sense: 5’-cgucacugaugucaacgacaatt-3’, antisense: 5’-uugucguugacaucagugacgtt-3’; DCHS1siRNA#2 sense: 5’-ccacccauauuugagcaacuatt-3’, antisense: 5’-uaguugcucaaauauggguggtt-3’; DCHS1siRNA#3 sense: 5’-gcucagagauugcacagguaatt-3’, antisense: 5’-uuaccugugcaaucucugagctt-3’. For DCHS1 overexpression, ISK or AN3CA cells were transfected with pcDNA3.1 (Vector group) or pcDNA3.1-DCHS1 (DCHS1OV). After 48 h, cells were collected for further research.

### Cell proliferation and colony formation assay

ISK or AN3CA cells transfected with siRNA or plasmids were seeded into 96-well plate at 3,000 cells/well. After 24, 48, 72 and 96 h, the absorptivity was measured at 450 nm by using the CCK-8 kit (RM02823, Abclonal, Wuhan, China). Cells transfected with DCHS1 siRNA were seeded into a 6-well plate. After 14 d, cells were fixed with methanol for 15 min and stained with 0.1% crystal violet for 30 min.

### Cell invasion assay

A 24-well Transwell chamber (8 μm aperture, Corning Costar, USA) was inoculated with 160 µL of cell suspension containing 5 × 10^4^ cells in serum –free medium. The lower chamber was completed immersed in a culture medium (800 µL) containing 10% fetal bovine serum. After 24 h incubation, cells were fixed with methanol for 15 min and stained with 0.1% crystal violet for 30 min. The migrated cells were counted under a microscope.

### RNA extraction and real-time quantitative PCR

Total RNA of tissues or cells was extracted with TRIzol reagent (Sangon Biotech, Shanghai, China). Quantitative RT-PCR was performed as previously described [25]. Primers for qRT-PCR were listed as follows: GAPDH forward, 5-’gccaaaagggtcatcatctc-3’ and reverse, 5-’gtagaggcagggatgatgttc-3’; DCHS1 forward, 5-’ggtacactgattggcgacatc-3’ and reverse, 5-’cccactgtgttcgtcaatgg-3’. The experiments were performed in triplicate with independent experiment samples.

### Western blot

Total protein extracts were prepared in radioimmunoprecipitation assay (RIPA) buffer (Beyotime, Shanghai, China) and the concentration of protein was measured by using a BCA kit (Beyotime, Shanghai, China). Equal amounts of protein were separated by SDS-PAGE, followed by electrophoretically transferred onto polyvinylidene difluoride membranes. After being blocked with 5% BSA at room temperature for 1 h, the membranes were incubated overnight at 4 °C with the following primary antibodies: E-cadherin (A3044, 1:1000), N-cadherin (A0433, 1:500), Vimentin (A2584, 1:500) and GAPDH (AC001, 1:10,000) (Abclonal, Wuhan, China). After being washed, the membranes were incubated with the corresponding secondary antibody for 2 h at room temperature. GAPDH was used as the loading control. The signals were visualized by using an enhanced chemiluminescence system (Abclonal, Wuhan, China).

### Statistical analysis

Statistical analysis was conducted using GraphPad Prism (version 8.0) and R (version 4.0.5). The t-test or Wilcoxon rank-sum test was used to compare the data of two groups. *P* < 0.05 was considered statistically significant.

**Table 1:** List of cancer types

Abbreviation	Cancer Types
ACC	Adrenocortical carcinoma
ALL	Acute Lymphoblastic Leukemia
BLCA	Bladder urothelial carcinoma
BRCA	Breast invasive carcinoma
CESC	Cervical squamous cell carcinoma and endocervical adenocarcinoma
CHOL	Cholangiocarcinoma
COAD	Colon adenocarcinoma
ESCA	Esophageal carcinoma
GBM	Glioblastoma
HNSC	Head and neck squamous cell carcinoma
KICH	Kidney chromophobe
KIRC	Kidney renal clear cell carcinoma
KIRP	Kidney renal papillary cell carcinoma
LAML	Acute myeloid leukemia
LUAD	Lung adenocarcinoma
LUSC	Lung squamous cell carcinoma
OV	Ovarian serous cystadenocarcinoma
PAAD	Pancreatic adenocarcinoma
PCPG	Pheochromocytoma and paraganglioma
PRAD	Prostate adenocarcinoma
SKCM	Skin Cutaneous Melanoma
STES	Stomach and Esophageal carcinoma
TGCT	Testicular Germ Cell Tumors
THCA	Thyroid carcinoma
UCEC	Uterine corpus endometrial carcinoma
UCS	Uterine carcinosarcoma
WT	High-Risk Wilms Tumor

## Results

### The different expression profiles of DCHS1 in human pan-cancer

**Fig. 1:**
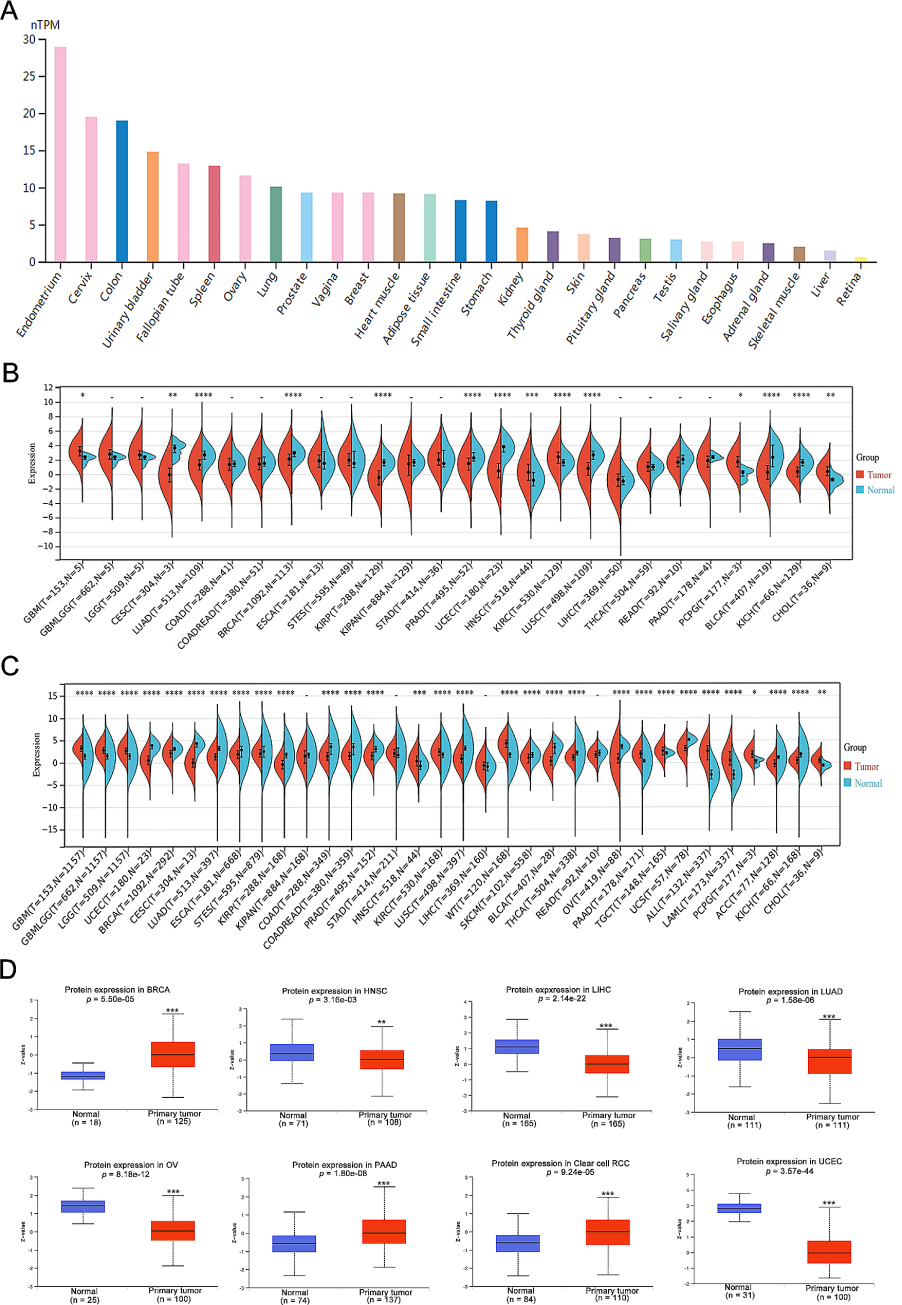
The expression levels of DCHS1 in various normal tissues and pan-cancer. **A**. Expression level of DCHS1 in normal tissues (HPA + GTEx datasets). **B**. The mRNA expression level of DCHS1 in different tumor tissues and corresponding normal tissues from TCGA datasets. **C**. The mRNA level of DCHS1 in different tumor tissues and corresponding normal tissues from TCGA and GTEx datasets. **D**. The protein levels of DCHS1 in pan-cancer analyzed by UALCAN database. *p < 0.05, **p < 0.01, ***p < 0.001

**Fig. 2:**
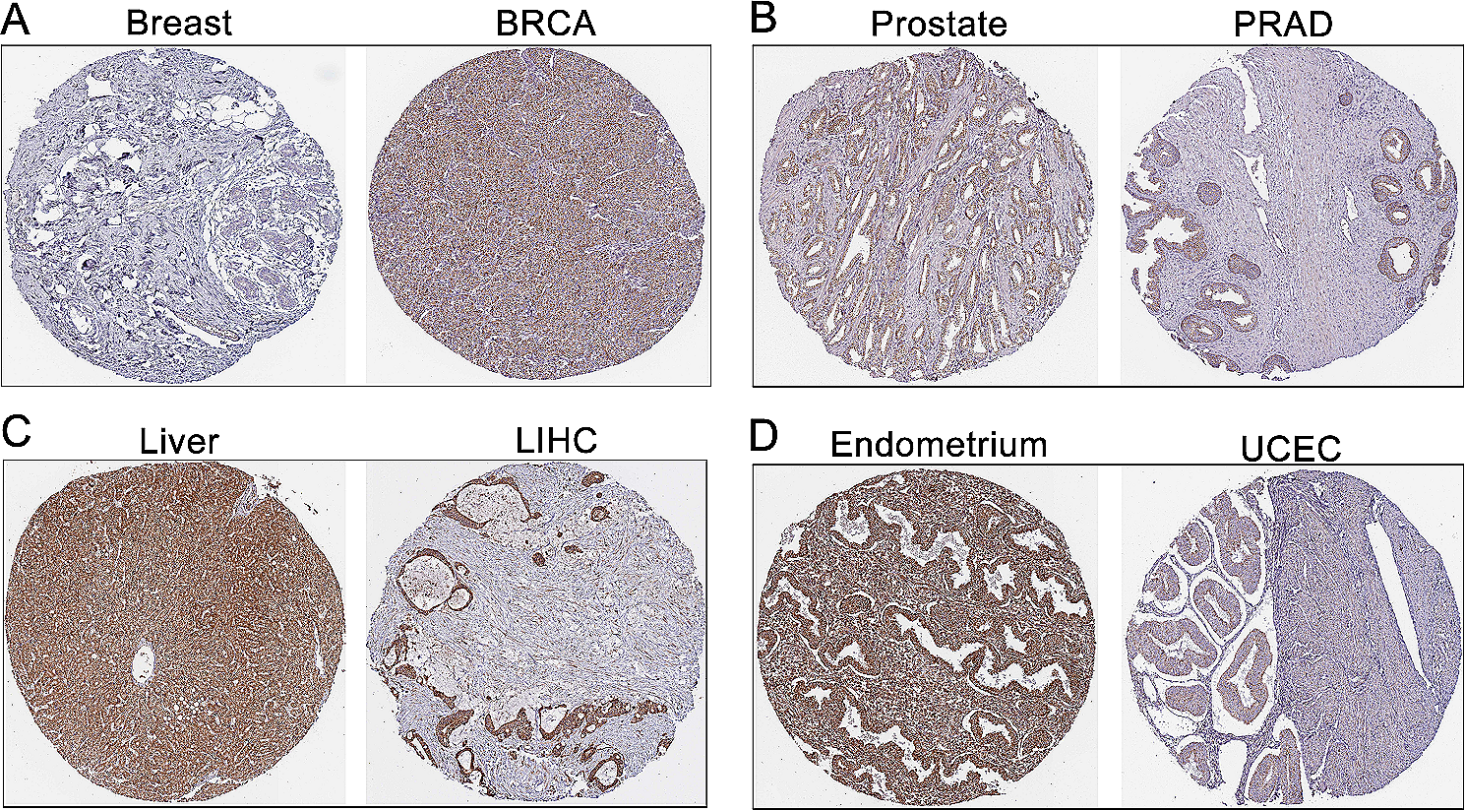
The pathology of DCHS1 protein expression profiles in normal tissues and BRCA (**A**), PRAD (**B**), LIHC (**C**) and UCEC (**D**) provided by HPA.

We examined DCHS1 expression in human normal tissues by using the GTEx dataset. In Fig. 1A, DCHS1 was overexpressed in Endometrium, Cervix, Colon and Urinary bladder tissues. Next, we further investigated the expression of DCHS1 in pan-cancer through the RNA-seq data of TCGA (Fig. 1B; Table 1). DCHS1 expression was overexpressed in GBM, HNSC, KIRC, PCPG, and CHOL, while downregulated in CESC, LUAD, BRCA, KIRP, PRAD, UCEC, LUSC, BLCA and KICH. Further expanded the normal sample size (TCGA + GTEx) (Fig. 1C), we found that DCHS1 was highly expressed in GBM, Glioma (GBMLGG), Brain Lower Grade Glioma (LGG), HNSC, KIRC, WT, PAAD, TGCT, ALL, LAML, PCPG and CHOL, while underexpressed in UCEC, BRCA, CESC, LUAD, ESCA, STES, KIRP, COAD, Colon adenocarcinoma/Rectum adenocarcinoma Esophageal carcinoma (COADREAD), PRAD, LUSC, SKCM, BLCA, THCA, OV, UCS, ACC and KICH. Then, we further analyzed the protein levels of DCHS1 by using UALCAN database. As shown in Fig. 1D, the protein levels of DCHS1 were significantly upregulated in BRCA, PAAD and clear cell RCC, while reduced in HNSC, LIHC, LUAD, OV and UCEC. Meanwhile, we employed the immunohistochemistry results from HPA database to confirm the intensity of DCHS1 immunohistochemical staining in cancers. The staining intensity of DCHS1 was greater in BRCA and PRAD than that in normal tissues, while the IHC staining of DCHS1 was weaker in LIHC and UCEC compared to normal tissues (Fig. 2).

### Prognostic and diagnostic values of DCHS1 in pan-cancer

**Fig. 3:**
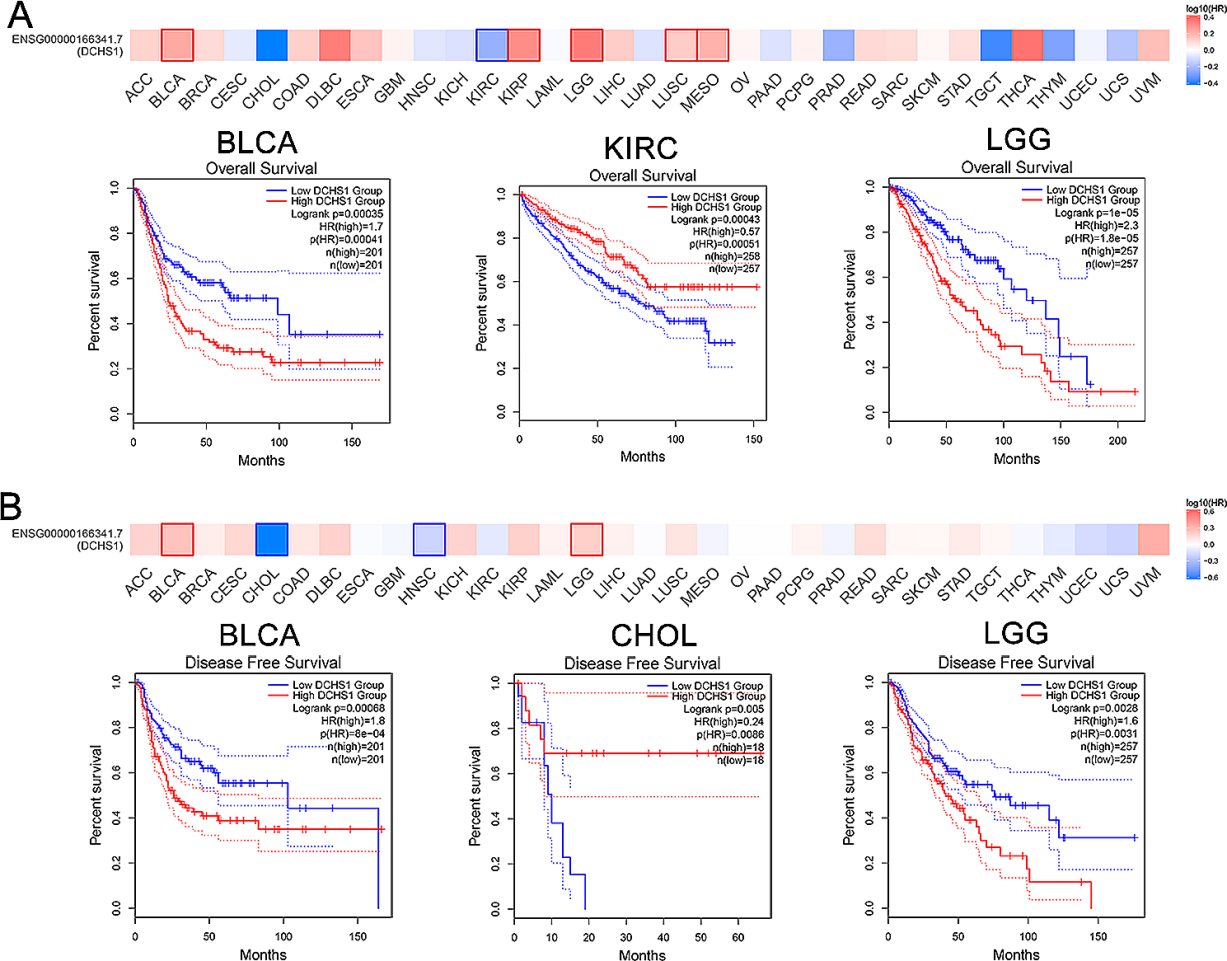
Prognostic values of DCHS1 in pan-cancer. The effect of DCHS1 gene expression on the patients’ overall survival (OS, **A**) and disease free survival (RFS, **B**) in pan-cancer analyzed by using GEPIA2

In order to evaluate the values of DCHS1 on patients’ prognosis, we performed Cox proportional hazards model and Kaplan-Meier analysis to analyze the relationship between DCHS1 expression and patients’ survival. DCHS1 high expression was associated with poor OS and RFS in BLCA (*p* = 0.00035) and LGG (*p* = 1e-05) (Fig. 3A), while it was correlated with good OS in KIRC (*p* = 0.00043) and good RFS in CHOL (*p* = 0.005) (Fig. 3B).

**Fig. 4:**
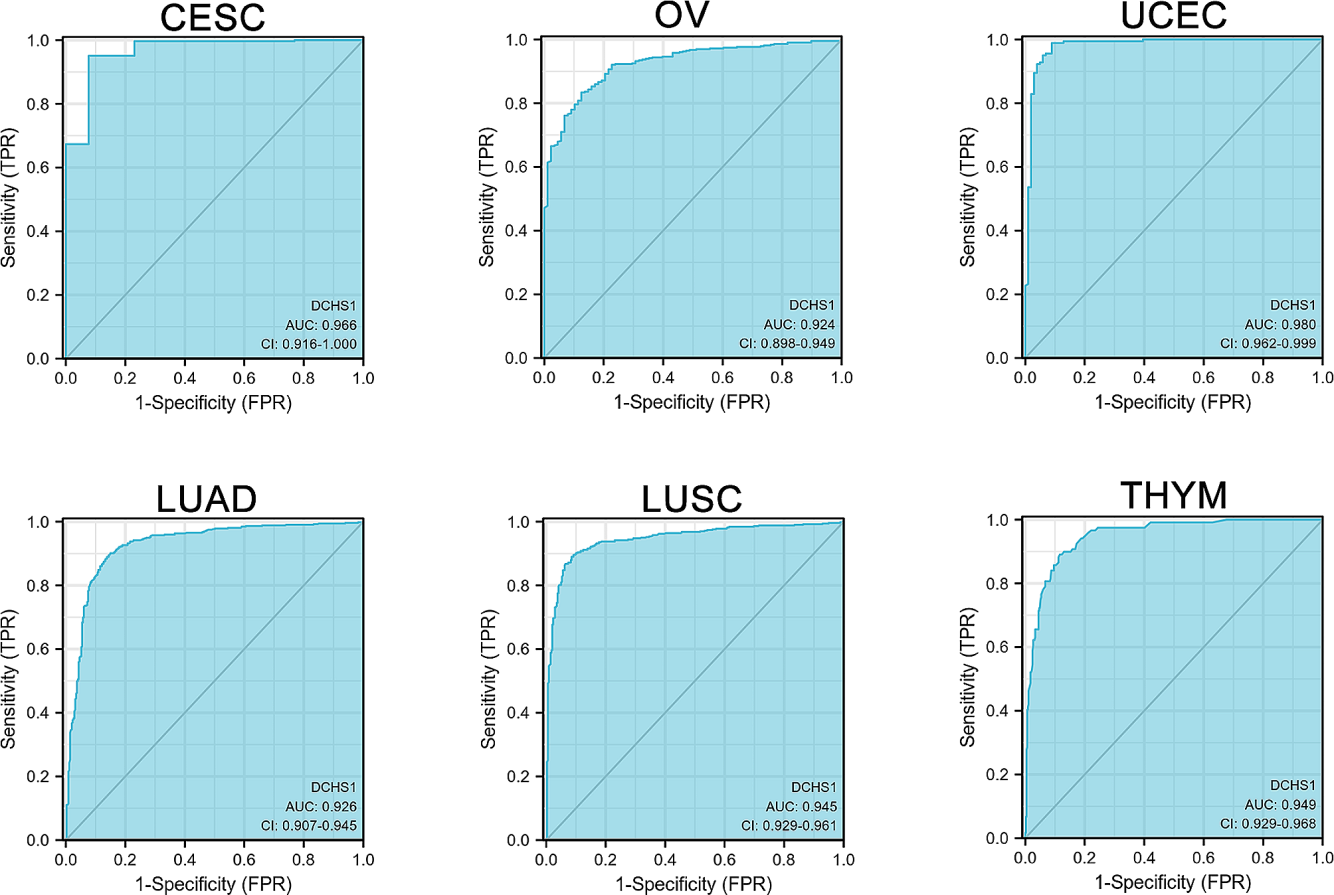
The ROC curve of diagnosis to distinguish tumor from normal tissues

To analyze the diagnostic value of DCHS1, we employed R package to conducted ROC curve analysis. The diagnostic values of DCHS1 gene expression with AUC values of 0.966 for CESC, 0.924 for OV, 0.980 for UCEC, 0.926 for LUAD, 0.945 for LUSC and 0.949 for THYM indicated high diagnostic values in these cancers (Fig. 4). The area was 0.893 in BLCA, 0.796 in BRCA, 0.858 in CHOL, 0.814 in COAD, 0.717 in KIRC, 0.841 in KIRP, 0.866 in PAAD, 0.835 in PRAD, 0.819 in READ and 0.814 in THCA (Figure S1).

### Genetic alteration analysis of DCHS1 in pan-cancer

**Fig. 5:**
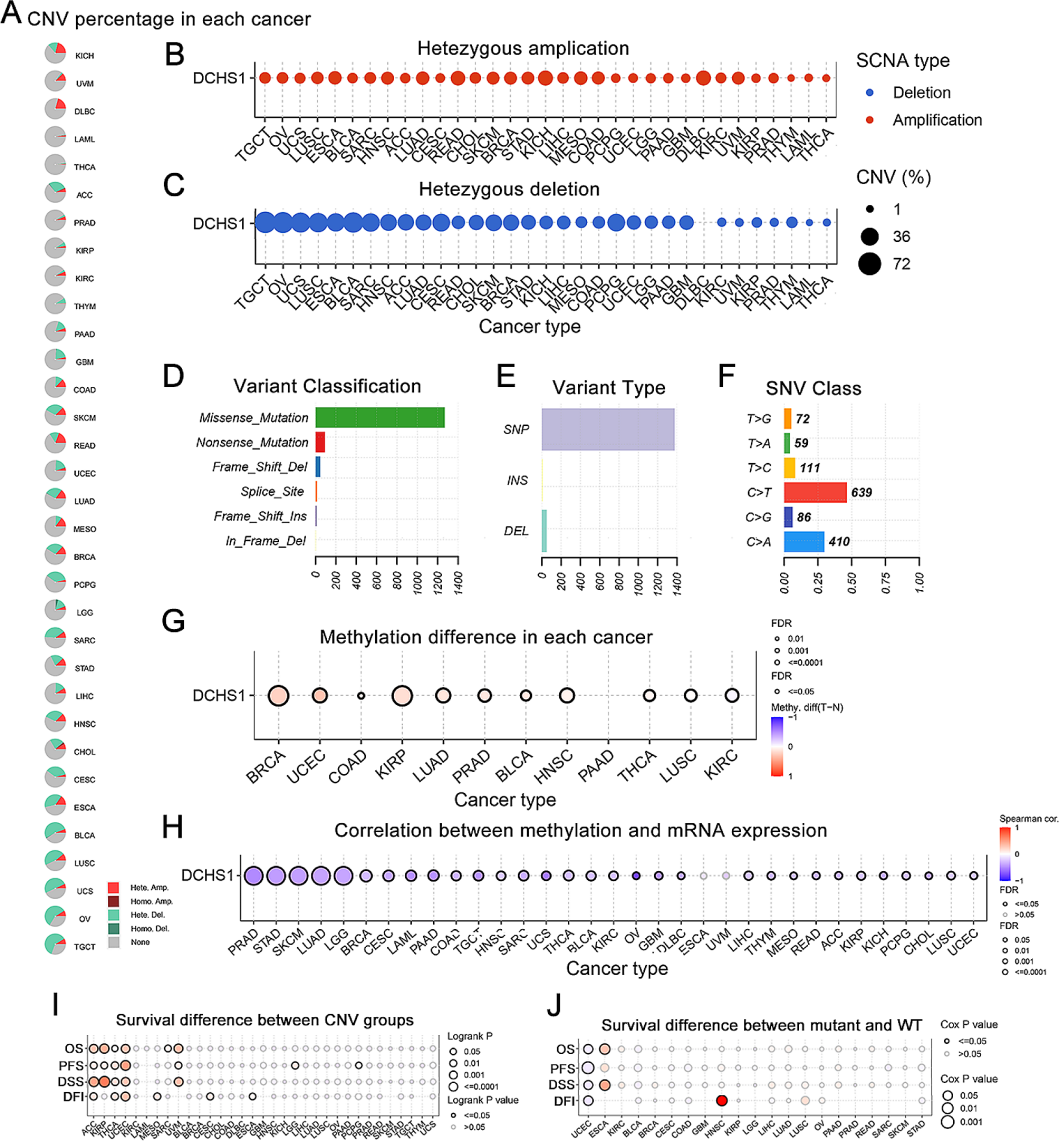
The genetic alterations and DNA methylation of DCHS1 in pan-cancer. **A**. Copy number variation (CNV) of DCHS1 in pan-cancer. **B-C**. The profiles of heterozygous amplification and deletion CNV of DCHS1 in pan-cancer. **D-F**. The single nucleotide variation (SNV) classes of DCHS1 in pan-cancer. **G**. The methylation difference between tumor and normal samples of DCHS1 in pan-cancer. **H**. The correlation between methylation and DCHS1 mRNA expression in pan-cancer. **I**. The difference of survival between CNV and wild type in pan-cancer. **J**. The survival difference between mutant and wild type in pan-cancer

To explore the genetic alteration of DCHS1 in pan-cancer, we used the GSCA database to analyze genetic alterations and DNA methylation. The results showed that CNV of DCHS1 occurred frequently in most cancers, including KICH, UVM, DLBC, ACC, PAAD, GBM, COAD, SKCM, READ, UCEC, LUAD, MESO, BRCA, PCPG, LGG, SARC, STAD, LIHC, HNSC, CHOL, CESC, ESCA, BLCA, LUSC, UCS, OV and TGCT, while only in LAML, THCA, PRAD, KIRP, KIRC and THYM, its frequency was low (Fig. 5A). In addition, the hetezyous amplication and deletion analysis showed that hetezygous amplication occurred frequently in READ, KICH and DLBC, and heterozygous deletions were common in TGCT, OV and BLCA (Fig. 5B-C). In Fig. 5D-F, we found that missense mutations occurred most frequently in DCHS1. Methylation analysis showed that significant difference in methylation levels was observed in BRCA, UCEC, KIRP, LUAD, PRAD, BLCA, HNSC, THCA, LUSC and KIRC (Fig. 5G). In Fig. 5H, significant negative correlation between methylation and DCHS1 expression was found in PRAD, STAD, SKCM, LUAD and LGG. Moreover, the prognostic analysis results indicated that the OS and DSS difference between CNV groups was mainly observed in KIRP, ACC, UCEC and UVM, and PFS and DFI survival difference was in UCEC (Fig. 5I). In Fig. 5J, the survival difference between SNV mutation and wild type was mainly observed in UCEC, ESCA and HNSC.

### Immune infiltration analysis of DCHS1

**Fig. 6:**
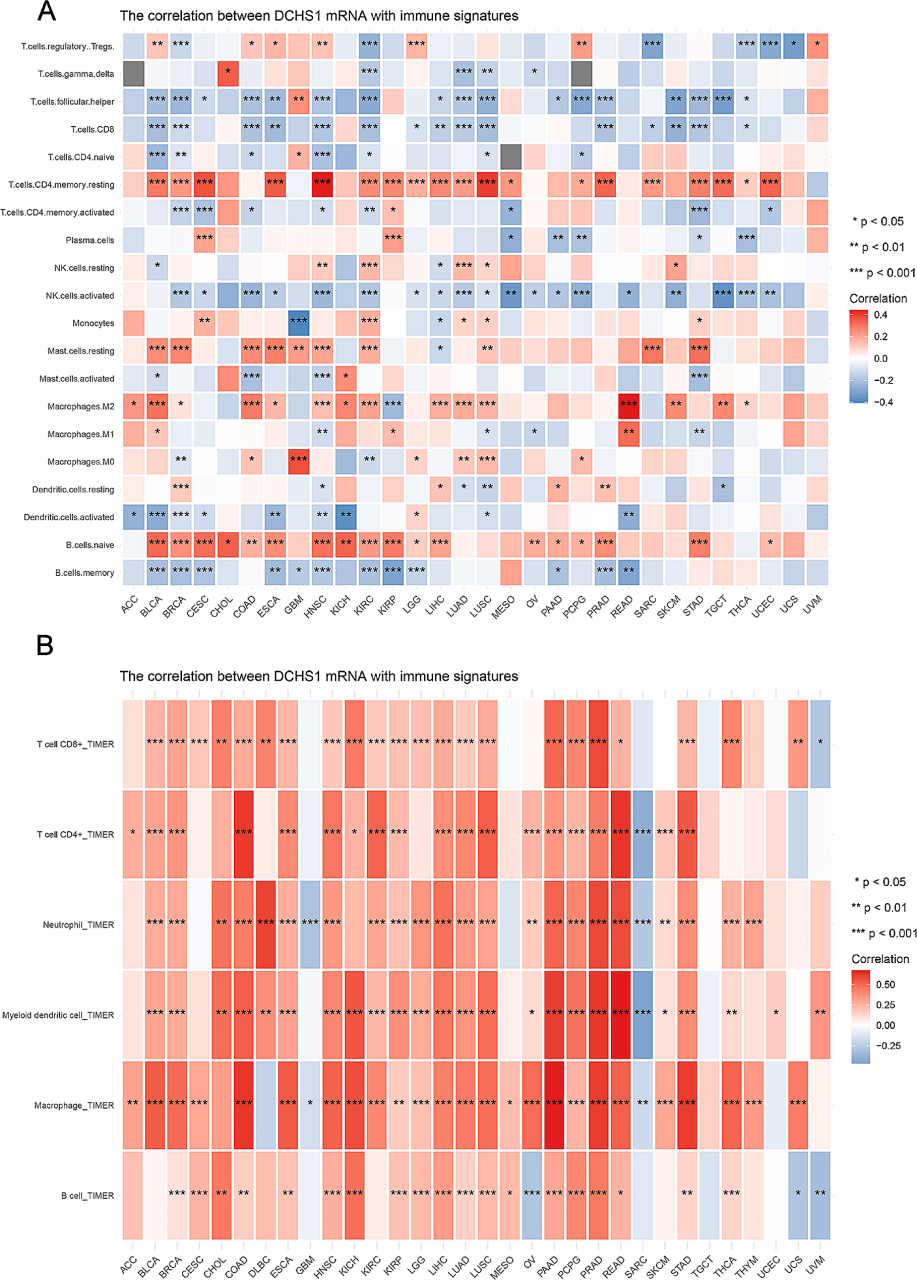
Association between DCHS1 and immune modulators in pan-cancer. **A**. The relationship between DCHS1 profile and immune signature. **B**. The correlation between DCHS1 expression and tumor immune infiltration. *p < 0.05, **p < 0.01, ***p < 0.001

We analyzed the correlation between DCHS1 expression and immune signature / tumor immune cell infiltration in the TCGA database by using UCSCXenaShiny online database. In Fig. 6A, DCHS1 expression was negatively associated with T cells follicular helper, T cells CD8, NK cells activated, Dendritic cells activated and B cells memory, however positively associated with T cells CD4 memory resting, Mast cells resting, Macrophages M2 and B cells naive. Figure 6B showed the correlation between DCHS1 expression and tumor cell infiltration. There were significant positive correlations with T cell CD8 + cells in 23 types of cancer, with CD4 + cells in 20 types of cancer, with Neutrophil in 24 types of cancer, with Myeloid dendritic cell in 25 types of cancer, with Macrophage in 27 types of cancer, with B cell in 22 types of cancer.

**Fig. 7:**
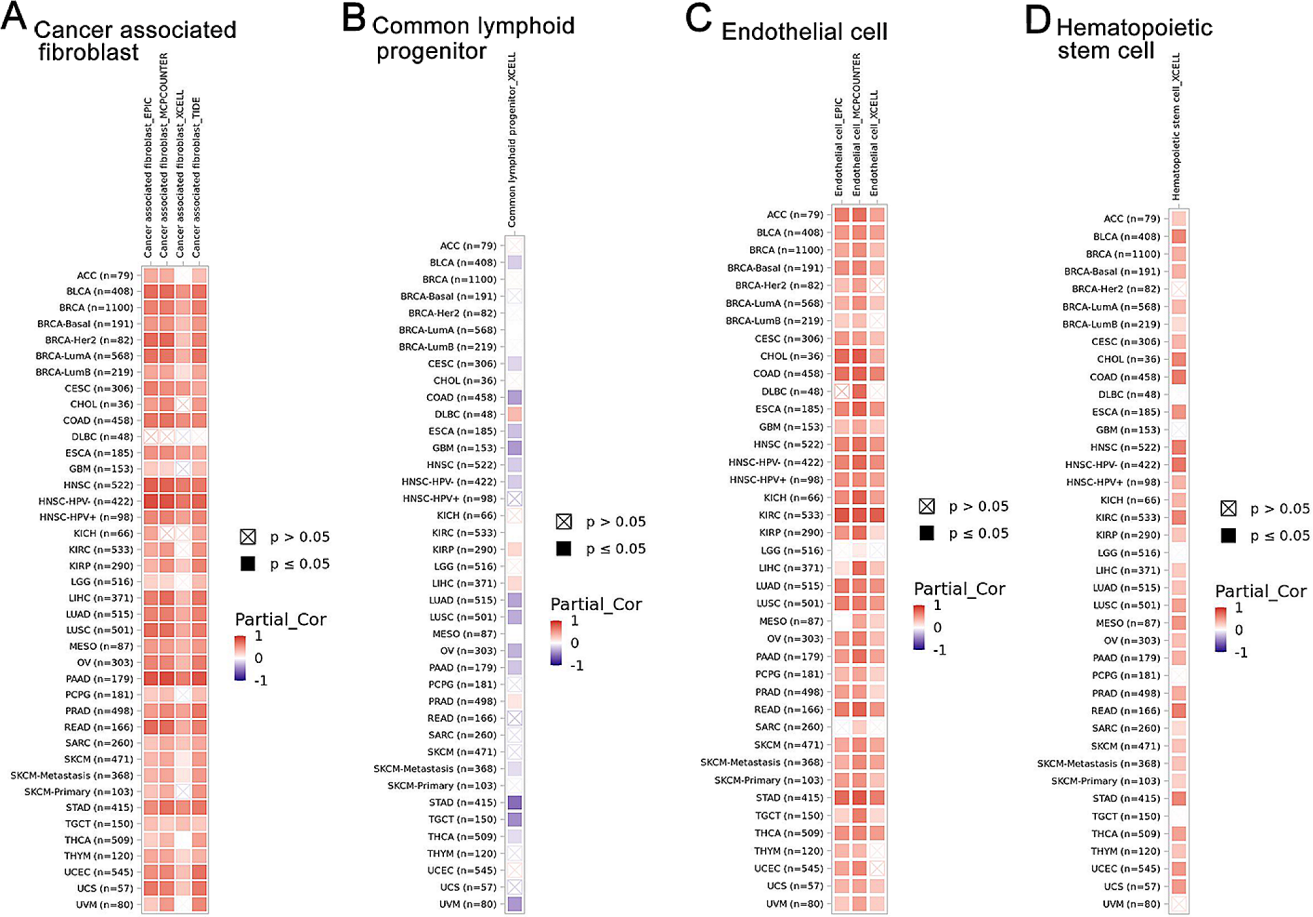
The correlation between DCHS1 expression and immune cells in pan-cancer. TIMER2.0 was used to analyze the relationship between DCHS1 expression and cancer associated fibroblast (**A**), Common lymphoid progenitor (**B**), Endothelial cell (**C**) and Hematopoietic stem cell (**D**)

We further analyzed the association between DCHS1 expression and various immune infiltrates in human cancers by using TIMER2.0. Our results displayed that DCHS1 expression was positively correlated with the immune infiltration of cancer associated fibroblast (CAF), Endothelial cell (EC), and Hematopoietic stem cell in most cancers (Fig. 7A). However, the negative correlation between DCHS1 expression and common lymphoid progenitor was found in GBM, STAD and TGCT (Fig. 7B). There were no significant correlations between DCHS1 expression and B cell, DC, Macrophage, Monocyte, Neutrophil, NK cell, T cell CD4 + and Treg (Figure S2).

**Fig. 8:**
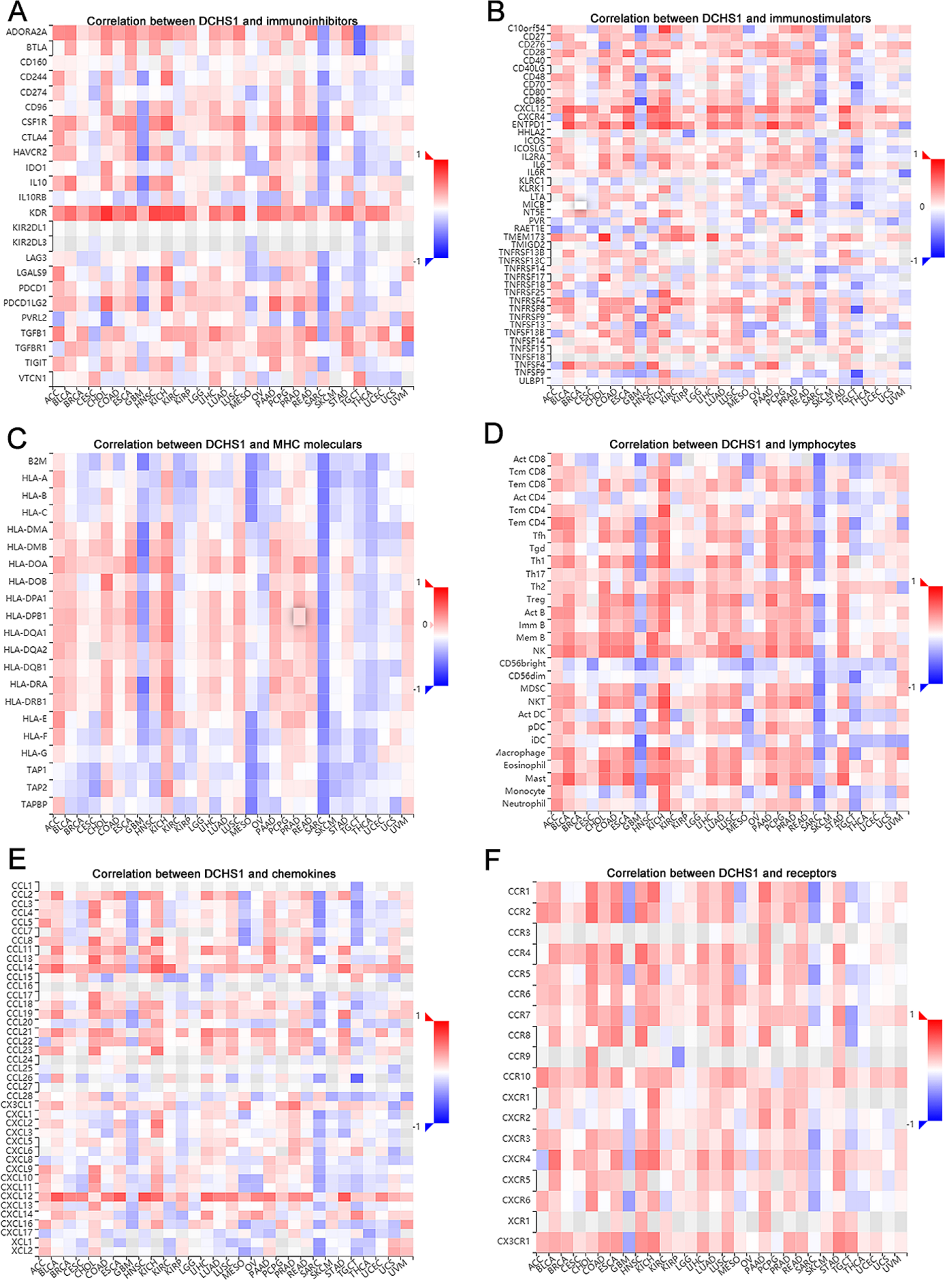
Relationship between DCHS1 expression and immuno-related genes in pan-cancer. The association between DCHS1 expression and immunoinhibitors (**A**), immunostimulators (**B**), MHC molecules (**C**), lymphocytes (**D**), chemokines (**E**), and receptors (**F**)

Subsequently, we analyzed the relationship between DCHS1 expression and Lymphocyte, immunomodulator, immunostimulators, MHC molecules and chemokine receptors in pan-cancer (Fig. 8). We found that the majority of immune-related genes in GBM, MESO and SARC showed a negative association, however, a positive relationship was observed in KICH and STAD.

### Stemness, TMB, MSI and drug sensitivity analysis

**Fig. 9:**
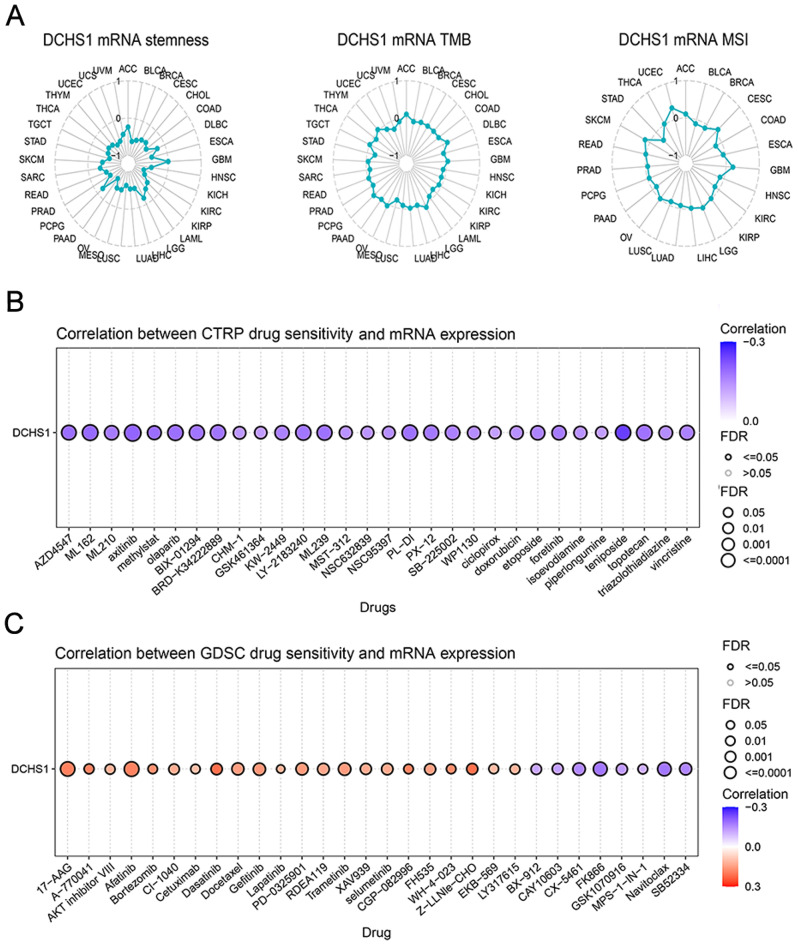
The correlation between DCHS1 expression and stemness, tumor mutational burden (TMB), microsatellite instability (MSI) and drug sensitivity. **A**. Relationship between DCHS1 and stemness, TMB and MSI analyzed by UCSCXenashiny. **B-C**. Drug sensitivity analysis based on DCHS1 expression using The Cancer Therapeutics Response Portal (CTRP) and Genomics of Drug Sensitivity in cancer (GDSC) databases

DCHS1 expression was significantly correlated with stemness in 31 types of cancer, including BLCA, BRCA, CESC, COAD, ESCA, HNSC, KICH, KIRC, KIRP, LAML, LGG, LIHC, LUAD, LUSC, MESO, OV, PAAD, PRAD, READ, SARC, SKCM, STAD, TGCT, THCA, THYM, UCEC, UCS, UVM, CHOL, PCPG and ACC, TMB only in STAD, KIRP, LGG, THCA, HNSC and LUSC, MSI in STAD, BRCA, COAD, KIRC, HNSC, GBM, BLCA, and LUSC (Fig. 9A). The potential relationship between DCHS1 expression and drug sensitivity was detected by using GSCA in two different databases (CTRP and GDSC). The CTRP database showed that DCHS1 expression was negatively correlated with most drug sensitivity including teniposide, axitinib and olaparib (Fig. 9B). According to the results in GDSC database, we found that DCHS1 expression was mainly positively correlated with 17-AAG, Atatinib and Trametinib, while negatively associated with FK866, Navitoclax and CX-5461 (Fig. 9C).

### Functional enrichment analysis of DCHS1

**Fig. 10:**
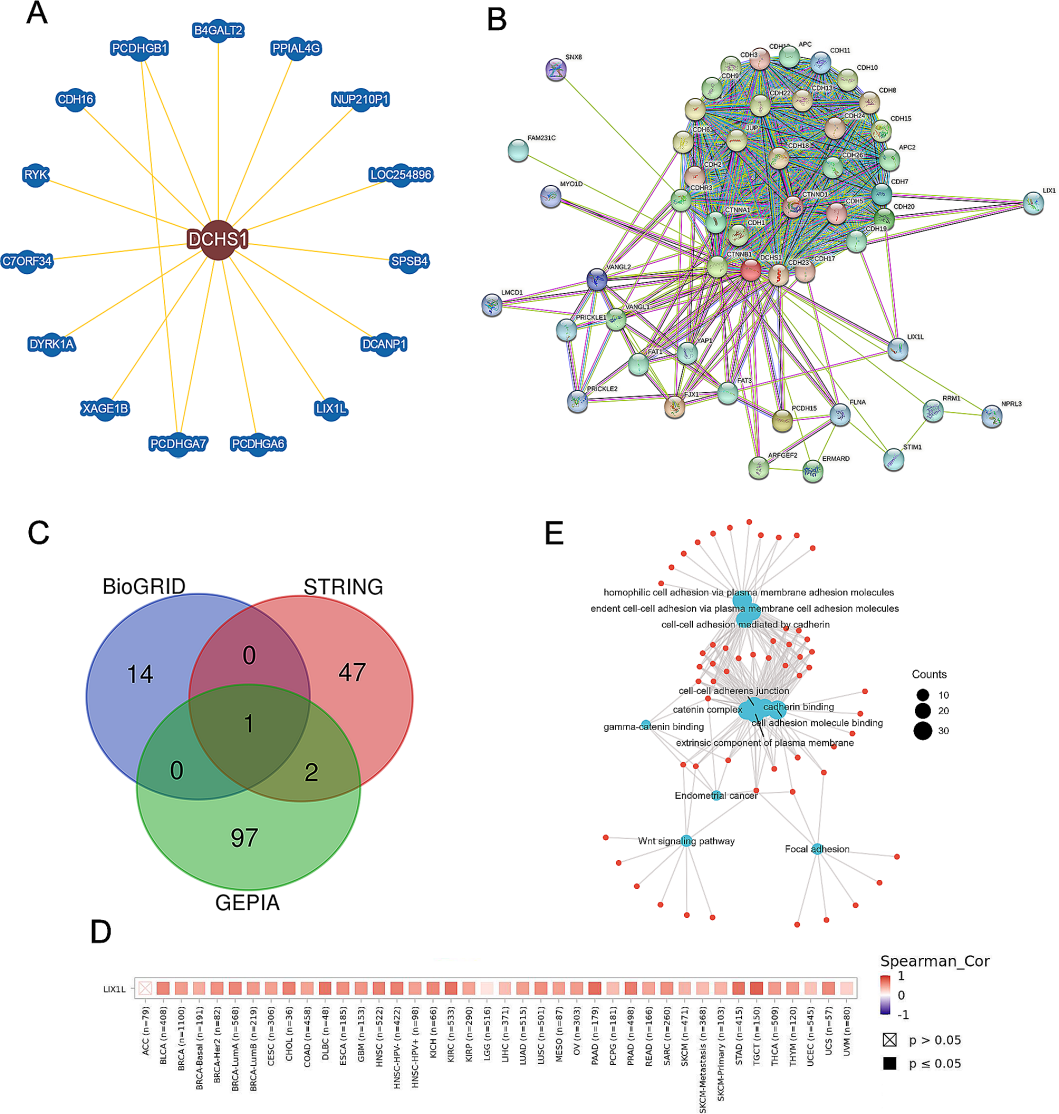
Functional enrichment analysis of DCHS1-related genes. **A**. 15 DCHS1-related proteins from BioGRID database. **B**. 50 DCHS1-interacted proteins from STRING database. **C**. The overlap among the proteins from BioGRID, STRING and GEPIA2.0. **D**. The correlation between DCHS1 and LIX1L analyzed by TIMER2.0. E. GO and KEGG enrichment analysis of DCHS1-related genes from BioGRID, STRING and GEPIA2.0

**Fig. 11:**
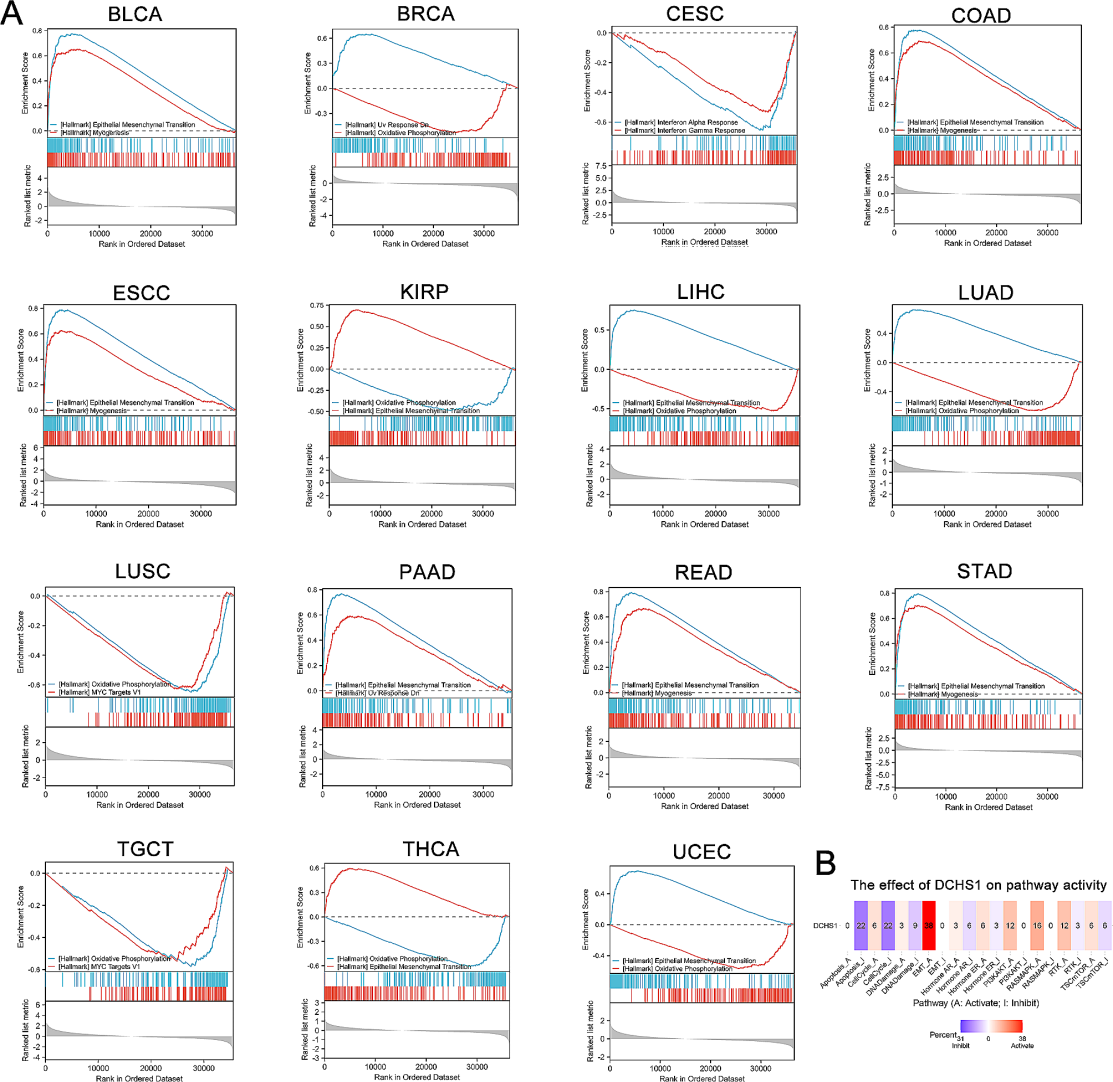
GSEA results of signaling pathways enriched in the high- and low-DCHS1 expression groups in pan-cancer (**A**). (**B**). GSCA was used to analyze the effect DCHS1 expression on pathway activity

We obtained 15 interacting proteins with DCHS1 from BioGRID web tool, including PCDHGB1, B4GALT2, PPIAL4G, NUP210P1, LOC254896, SPSB4, DCANP1, LIX1L, PCDHGA6, PCDHGA7, XAGE1B, DYRK1A, C7ORF34, RYK and CDH16 in Fig. 10A. A total of 50 DCHS1-interacted proteins were obtained from STRING web (Fig. 10B). The top 100 DCHS1 co-expressed genes were selected from GEPIA2.0. An intersection analysis of the above three groups (BioGRID, STRING and GEPIA2.0) showed only one common member, namely, LIX1L (Fig. 10C). By using TIMER2.0, we analyzed the correlation between DCHS1 and LIX1L in human cancer and found that DCHS1 expression was significantly positively correlated with LIX1L in pan-cancer (Fig. 10D). Then we combined the above three datasets to performed GO/KEGG enrichment analysis. In Fig. 11E, KEGG data indicated that DCHS1-related genes might be involved in Wnt signaling pathway, endometrial cancer pathogenesis and Focal adhesion. GO enrichment analysis data showed that most of these genes were closely linked with the biological process, including in hemophilic cell adhesion, cell-cell adhesion and calcium-dependent cell-cell adhesion (Fig. 10E).

We further employed GSEA to investigate the main biological process affected by DCHS1 in pan-cancer. In Fig. 11A-O, GSEA results revealed that Epithelial Mesenchymal Transition (EMT) pathway was more enriched in the high-risk group than in the low-risk group in BLCA, COAD, ESCC, KIRP, LIHC, LUAD, PAAD, READ, STAD, THCA and UCEC. While, the low status of DCHS1 was associated with the enrichment of oxidative phosphorylation pathway in BRCA, KIRP, LIHC, LUAD, THCA and UCEC. We further employed GSCA to analyze the effect DCHS1 expression on pathway activity in Fig. 11P. We also found that DCHS1 expression was significantly positively associated with EMT activation.

### DCHS1 is involved in cell proliferation and migration in endometrial cancer cells

**Fig. 12:**
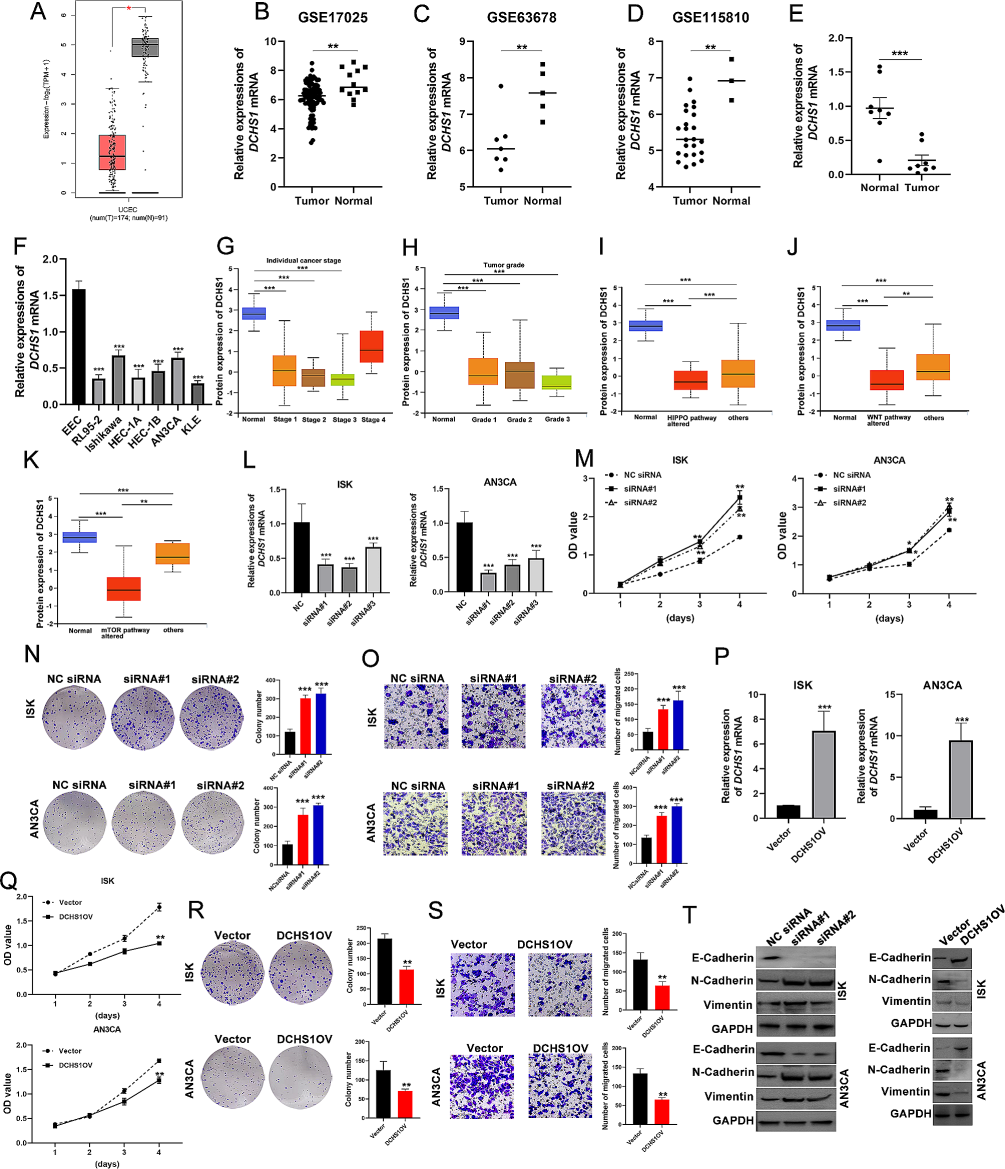
DCHS1 was involved in cell proliferation, migration and EMT. **A**. DCHS1 was downregulated in UCEC analyzed by GEPIA2.0. **B-D**. The differential expression of DCHS1 in GEO17025, GSE63678 and GSE115810. **E-F**. DCHS1 was lowly expressed in EC tissues and cell lines analyzed by qRT-PCR. Total RNA was isolated from 8 pairs of UCEC tissues and cell lines. **G-H**. DCHS1 differential expression in individual cancer stages and tumor grades via UALCAN database. **I-K**. The relationship between DCHS1 expression and HIPPO, WNT and mTOR pathways by using UALCAN online database. **L**. The silencing efficiency of siRNAs transfection into ISK and AN3CA cells analyzed by qRT-PCR. M. CCK8 analysis of DCHS1 silencing in ISK and AN3CA. **N**. Colony formation assay of the effect of DCHS1 knockdown on proliferation. **O**. Transwell assay of the invaded cells after DCHS1 silencing. **P**. The overexpression efficiency of pcDNA3.1-DCHS1 and pcDNA3.1 transfection into ISK and AN3CA cells analyzed by qRT-PCR. **Q**. CCK8 analysis of DCHS1 overexpression in ISK and AN3CA. **R**. Colony formation assay of the effect of DCHS1 overexpression on proliferation. **S**. Transwell assay of the invaded cells after DCHS1 overexpression. **T**. WB detecting the effect of DCHS1 silencing or overexpression on EMT markers. *p < 0.05, **p < 0.01, ***p < 0.001

According to the above results, we found that DCHS1 was highly expressed in endometrium and downregulated in EC on mRNA and protein levels (Fig. 1). DCHS1 had high accuracy (AUC = 0.980) for the diagnosis of UCEC (Fig. 4). GO and KEGG enrichment analysis of DCHS1-related genes from BioGRID, STRING and GEPIA2.0 revealed that they were significantly enriched in endometrial cancer, Wnt signaling pathway and Focal adhesion (Fig. 10). We further investigated the function of DCHS1 in the context of UCEC. GEPIA2.0 analysis results showed that DCHS1 was significantly downregulated in UCEC (Fig. 12A). Similar results were found in the patients from GSE17025, GSE63678 and GSE115810 datasets in the GEO database (Fig. 12B-D). Based on these results, we further collected a total of 8 pairs of UCEC tissue samples and found that DCHS1 expression was considerably lower in UCEC tissues than normal tissues (Fig. 12E). Similarly, the mRNA levels of DCHS1 in UCEC cell lines (RL95-2, ISK, HEC-1 A, HEC-1B, AN3CA and KLE) were lower than those in EEC (Fig. 12F). By using UALCAN, we further analyzed DCHS1 protein based on individual cancer stages and tumor grades in Fig. 12G-H. Similarly, DCHS1 protein levels in normal group were significantly higher than those in four stages and three grades.

To explore the role of DCHS1 in UCEC, we investigated DCHS1 proteomic expression profiles based on various signaling pathways. The results showed the close relationships between DCHS1 protein expression and Hippo, Wnt, mTOR, RTK, P53/Rb-related, NRF2, myc/mycn pathways (Fig. 12I-K, Figure S3A-D). By using cBioPortal web tool, we examined the genetic alterations affecting DCH1 expression in UCEC and downloaded the mutations in Table S2. As shown in Figure S3E-F, the most frequently occurring genetic modification was mutation, especially missense mutations (169/191). Survival analysis revealed that the altered group of DCHS1 had better OS, PFS, DFS and DSS compared to the unaltered group (Figure S3G-J).

We transfected ISK and AN3CA cells with three siRNA and carried out qRT-PCR to examine the knockdown efficiency. In Fig. 12L, DCHS1 expression in three siRNA transfected groups was significantly downregulated than that in NC siRNA transfection group, and the transfection of siRNA#1 and siRNA#2 showing higher silencing efficiency. Thus, we chose siRNA#1 and siRNA#2 for further experiments. CCK8 results showed that the silencing of DCHS1 significantly enhanced the proliferation ability (Fig. 12M). In Fig. 12N, the colony numbers in DCHS1 siRNA-transfected cells were increased than those in control group. The transwell assay indicated that the migration ability of ISK and AN3CA cells was significantly improved after DCHS1 silencing in Fig. 12O. We further explored the effect of DCHS1 overexpression on cell proliferation and migration. The transfection of pcDNA3.1-DCHS1 significantly increased the expression of DCHS1 compared with pcDNA3.1 transfection (Fig. 12P). CCK8, colony formation and Transwell assays revealed that the overexpression of DCHS1 significantly blocked cell proliferation and migration (Fig. 12Q-S). These data reveal that DCHS1 was involved in cell proliferation and migration in UCEC.

According to the GSEA results and the relationship between DCHS1 expression and pathway activity (Figure S3K), we found that DCHS1 might play a potential role in EMT process in UCEC. Western blotting was employed to explore the relationship between DCHS1 expression and EMT marker, including E-cadherin, Vimentin and N-cadherin (Fig. 12T). The obtained results showed that the knockdown of DCHS1 resulted in a decrease in E-cadherin and an increase in the levels of N-cadherin and Vimentin. In the contrast, the overexpression of DCHS1 induced the upregulation of E-cadherin, but the downregulation of N-cadherin and Vimentin. These data showed that DCHS1 contributed to the EMT process in UCEC.

## Discussion

Protocadherins (PCDHs), belonging to the cadherin superfamily, are subdivided into clustered and non-clustered PCDHs [26]. PCDHs play a significant role in cell adhesion and signaling between cells. Some reports showed that PCDHs are dysregulated in various cancers, such as PCDH10 [27], PCDH17 [28] and PCDH8 [29]. DCHS1, also named PCDH16, is a member of cadherin superfamily and regulates cell polarity and proliferation via interacting with cadherin Fat [30]. Increasing evidences reported that dysregulation of cadherin expression contributes to tumor progression, such as the loss of E-cadherin expression or the gain of N-cadherin [31]. Although it is reported that mutated DCHS1 was frequently found in the aggressive papillary thyroid microcarcinomas, the biological functions of DCHS1 in tumors were limited. Here, we conducted a pan-cancer analysis to evaluate the roles of DCHS1 across multiple databases and identify its value in prognosis and diagnosis.

In our study, we found that DCHS1 expression was dysregulated in many cancers based on the pan-cancer analysis of TCGA and GTEx databases. And the similar results were observed on the protein levels of DCHS1. Genetic and epigenetic modifications mainly contribute to alter gene expression in cancer [32]. The accumulation of genetic alterations is thought to drive the progression of normal cells to cancer [4]. Epigenetic modifications play a crucial role in the progression of cancer by significantly influencing gene activity and cellular function, including DNA methylation and histone modifications (phosphorylation, ubiquitination, sumoylation and acetylation) [33]. In addition, TME plays a pivotal role in tumor initiation and progression by creating a dynamic interaction with cancer cells [34], and the cell components of TME are usually different in different cancers [35]. We speculated that the variation in DCHS1 expression across different cancer types might be the result of the combination of genetic changes and epigenetic modifications under heterogeneous TME. Furthermore, KM survival curves confirmed that DCHS1 might be a potential reliable biomarker in some cancers. For example, DCHS1 was significantly associated with some clinicopathologic features including OS and DFS. In BLCA and LGG, DCHS1 was identified as a risky factor and its high expression was associated with poor OS and DFS. However, DCHS1 acted as a favorable factor in KIRC and CHOL. ROC diagnostic curve indicated that DCHS1 might serve as a novel cancer biomarker for diagnostic application, especially in CESC, OV, UCEC, LUAD, LUSC, THYM. These results revealed the differential expression of DCHS1 in different cancers and the value of DCHS1 on prognosis and diagnosis.

TME is composed of diverse immune cells, CAFs, ECs and the extracellular matrix (ECM). Cells and factors of the TME regulate tumorigenesis, invasion and metastasis [36, 37]. Immunoinfiltration analysis indicated that DCHS1 expression was significantly correlated with the abundance of infiltrating immune cells, including CD8 + T, CD4 + T, Neutrophil, Myeloid dendritic cell, Macrophage and B cells. CAFs are the most essential components of TME, and they promote cancer cell proliferation, therapy resistance and immune exclusive by secreting growth factors, chemokines, cytokines, exosomes and other molecules [38]. In tumor tissues, CAFs interact with the cancer cells and immune cells to induce immune suppression and promote carcinoma progression and metastasis [39]. For these reasons, numerous studies have indicated that CAFs could be selected as an emerging target of anti-cancer immunotherapy [40]. However, recent study has revealed that CAF populations activated by stroma-specific hedgehog signaling inhibit tumor growth and invasion by blocking matrix stiffness [41]. The identification of a role of CAFs in restraining tumor progression has added a further layer of complexity of CAFs [42]. A new therapeutic strategy targeting CAFs is the “stromal switch”, in which tumor-promoting CAFs is shifted towards a quiescent or tumor-restraining phenotype. ECs are one of the main sources of CAFs and express increased levels of inhibitory immune checkpoint molecules to induce immunosuppression [43]. Tumor ECs express lower levels of adhesion molecules to impair barrier function and express higher levels of inhibitory immune checkpoint molecules to induce immunosuppression [44]. We found that DCHS1 expression is positively associated with CAFs and ECs in pan cancers. These data suggest that DCHS1 plays an important function in modulating the TME.

Multigen co-expression analysis and experiments were performed to validate the function of DCHS1 in UCEC. Experiments in vitro validated that DCHS1 was downregulated in UCEC tissues and cell lines, which was consistent with the results obtained from TCGA and GEO datasets. In the work, we found that the silencing of DCHS1 significantly improved cell proliferation, migration and EMT, and the overexpression of DCHS1 obviously blocked cell proliferation, migration and EMT. These results indicated that DCHS1 serves as an anticancer gene in the progression of UCEC.

Taken together, our study revealed that DCHS1 was differentially expressed in pan cancer, and it may serve as a potential tumor prognostic and diagnostic biomarker. The epigenetic alterations of DCHS1 are mainly hetezygous deletions and missense mutations. CAFs and ECs infiltration was significantly positively associated with DCHS1 expression. Our GSEA results indicated that DCHS1 expression was significantly linked with EMT pathway. Lastly, in vitro experiments showed that DCHS1 knockdown promoted cell proliferation, migration and EMT, and the overexpression of DCHS1 blocked cell proliferation, migration and EMT in UCEC. These findings revealed the role of DCHS1 in pan-cancer tumorigenesis and progression, and provided the basis for immunotherapy research in UCEC. However, there still were some limitations in our study. Firstly, in our work, we found the positive relationship between anti-tumor gene DCHS1 expression and CAFs and ECs. However, the mechanism of the interacting of DCHS1 expression and CAFs and ECs remains unknown. Secondly, we mainly explored the function of DCHS1 in UCEC in the context of cells. In vivo animal experiments need to be performed to deeply dig the role of DCHS1 in UCEC.

### Electronic supplementary material

Below is the link to the electronic supplementary material.

Supplementary Material 1

Supplementary Material 2

Supplementary Material 3

Supplementary Material 4

Supplementary Material 5

Supplementary Material 6

## Data Availability

The datasets generated and analyzed are mainly available from TCGA, GEO, HPA, UALCAN, STRING, GEPIA2 and cBioportal that provide free online tools and resources. Some datasets used and/or analyzed during the current study are available from the corresponding author on reasonable request.
