# Supplementary figures and images for: Dachsous cadherin related 1 (DCHS1) is a novel biomarker for immune infiltration and epithelial-mesenchymal transition in endometrial cancer via pan-cancer analysis
Source: J Ovarian Res. 2024 Aug 9;17:162. doi: 10.1186/s13048-024-01478-1 (PMC11312386; doi:10.1186/s13048-024-01478-1)

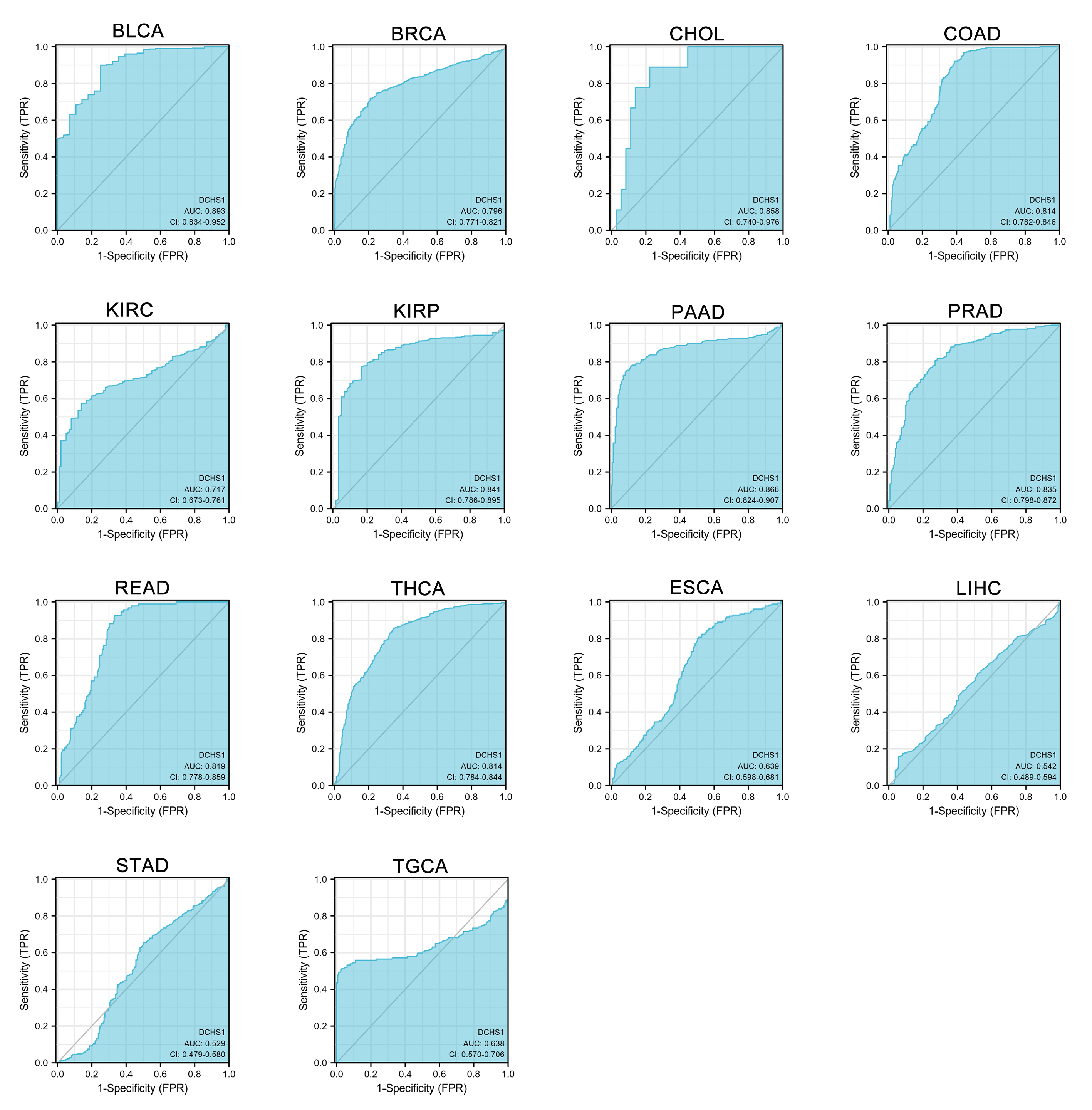

Supplement: Supplementary file 1 — Supplementary Material 1 [file 13048_2024_1478_MOESM1_ESM.tif]

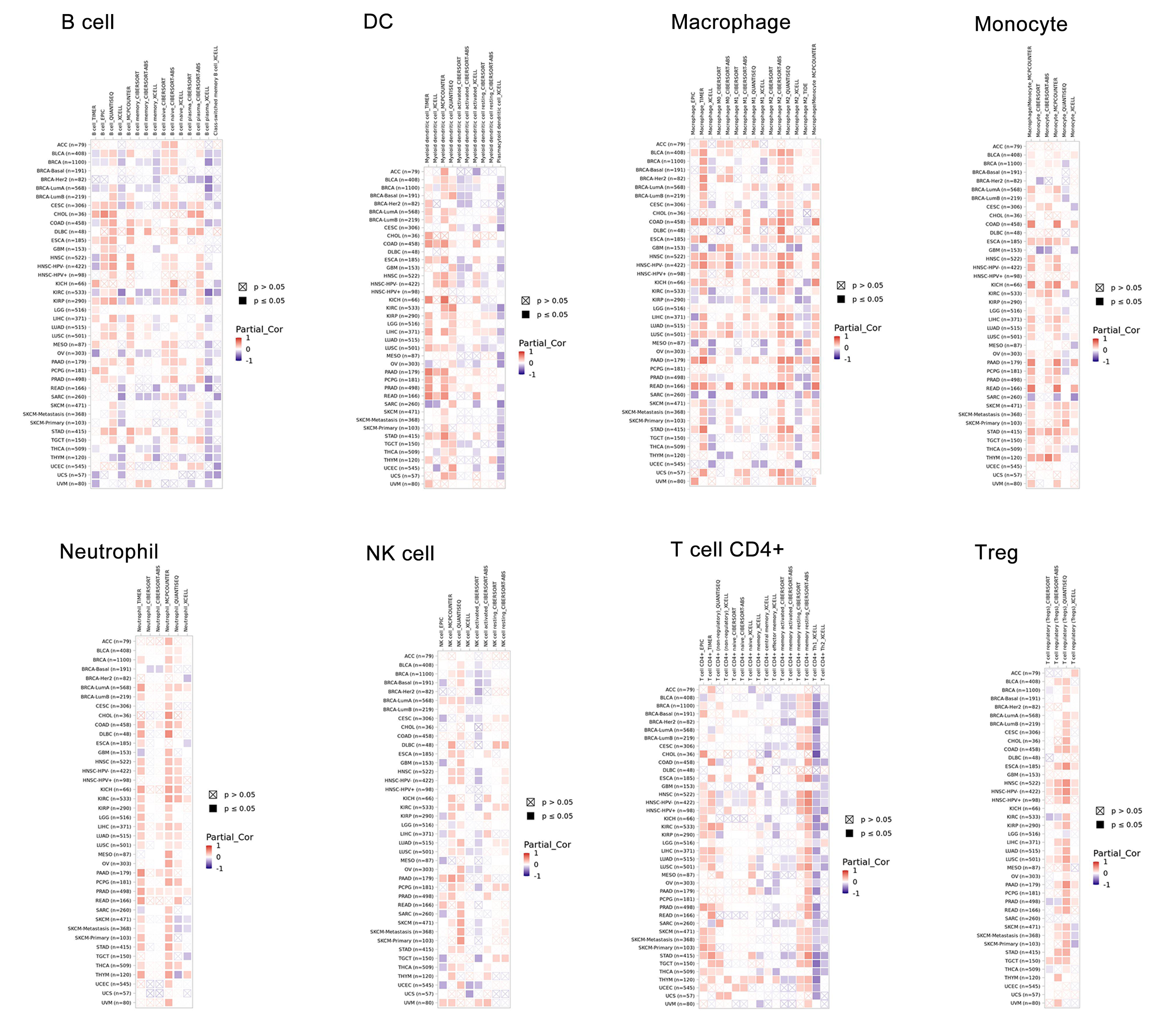

Supplement: Supplementary file 2 — Supplementary Material 2 [file 13048_2024_1478_MOESM2_ESM.tif]

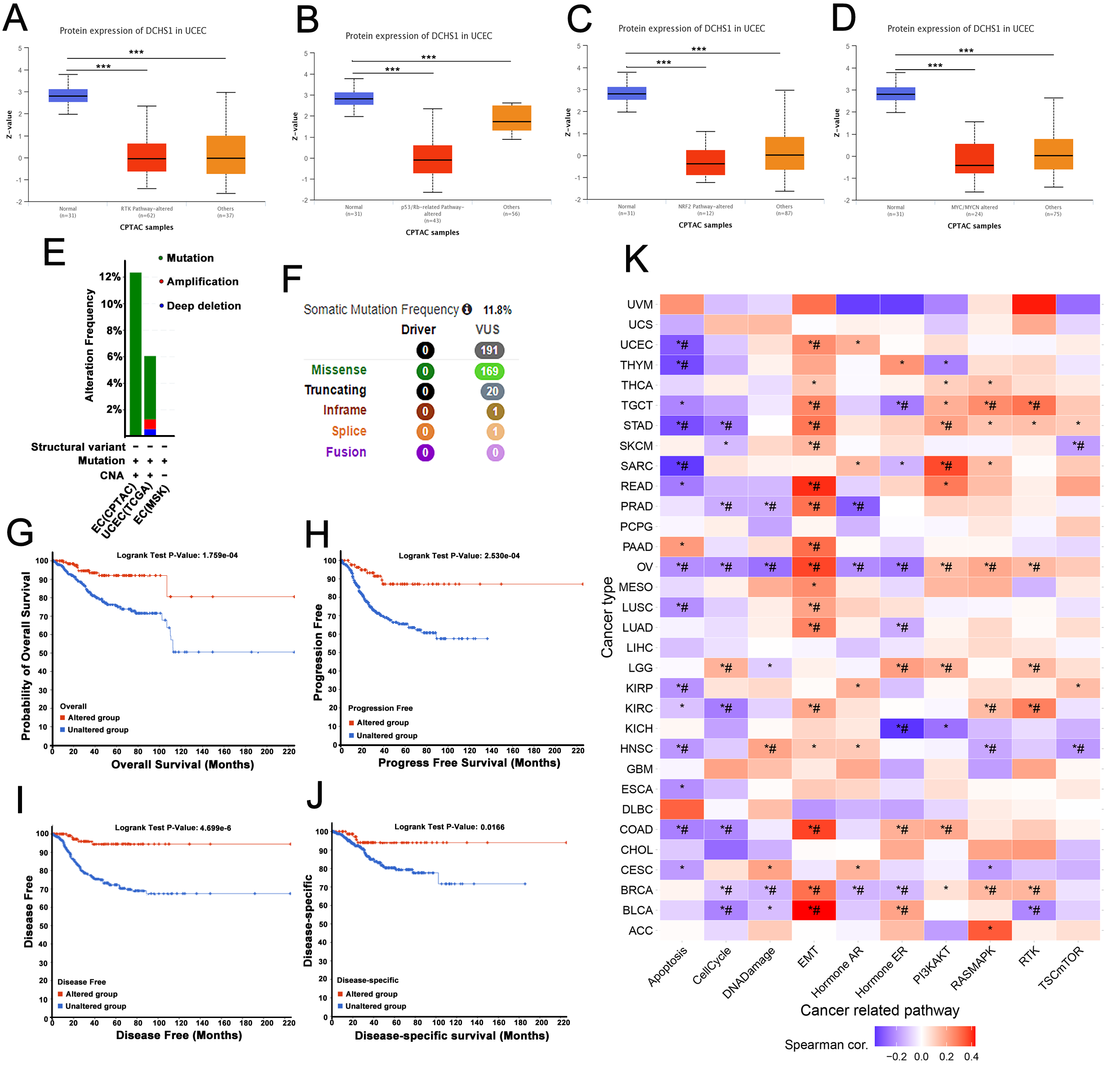

Supplement: Supplementary file 3 — Supplementary Material 3 [file 13048_2024_1478_MOESM3_ESM.tif]
