# Supplementary material for: Dachsous cadherin related 1 (DCHS1) is a novel biomarker for immune infiltration and epithelial-mesenchymal transition in endometrial cancer via pan-cancer analysis
Source: J Ovarian Res. 2024 Aug 9;17:162. doi: 10.1186/s13048-024-01478-1 (PMC11312386; doi:10.1186/s13048-024-01478-1)
Supplement: Supplementary file 4 — Supplementary Material 4 [file 13048_2024_1478_MOESM4_ESM.docx]

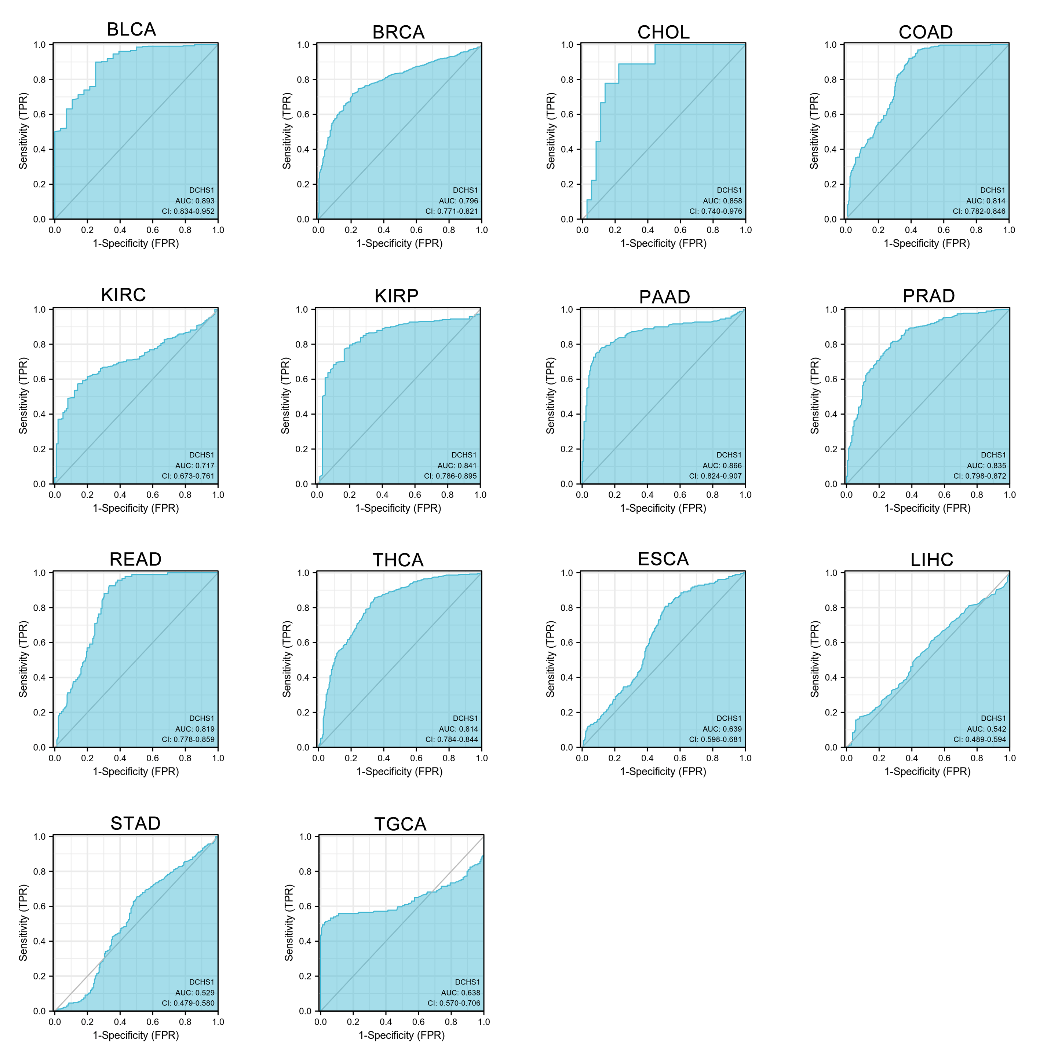


Figure S1. The ROC curve of diagnosis to distinguish tumor from normal tissues. AUC < 0.9.


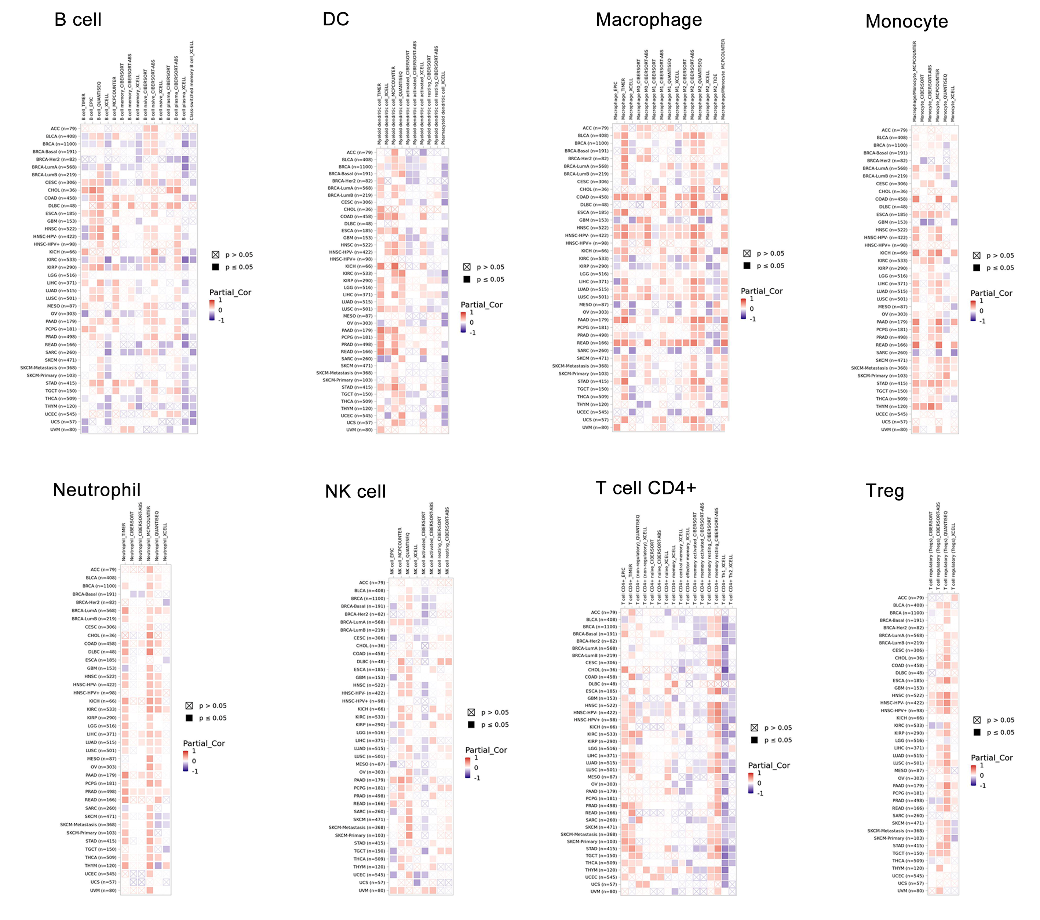


Figure S2. The correlation between DCHS1 expression and immune cells in pan-cancer.


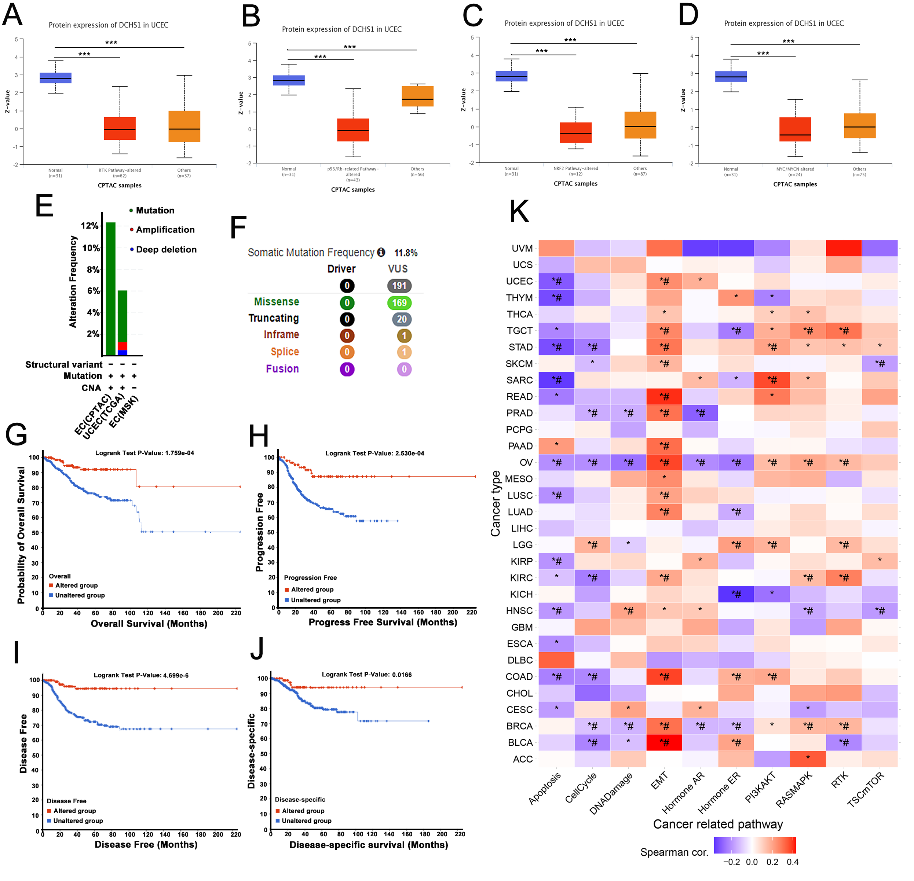


Figure S3. A-D. The correlation between DCHS1 expression and RTK, P53, NRF2 and MYC. E. The alteration frequency with different types of DCHS1 mutations in UCEC. F. The genetic modifications in UCEC. G-J. The survival analysis of altered group and unaltered group (OS, PFS, DFS and DSS). K. The relationship between DCHS1 and pathways.
