# Supplementary material for: Dachsous cadherin related 1 (DCHS1) is a novel biomarker for immune infiltration and epithelial-mesenchymal transition in endometrial cancer via pan-cancer analysis
Source: J Ovarian Res. 2024 Aug 9;17:162. doi: 10.1186/s13048-024-01478-1 (PMC11312386; doi:10.1186/s13048-024-01478-1)

ISK-DCHS1 RNAi-E-cadherin


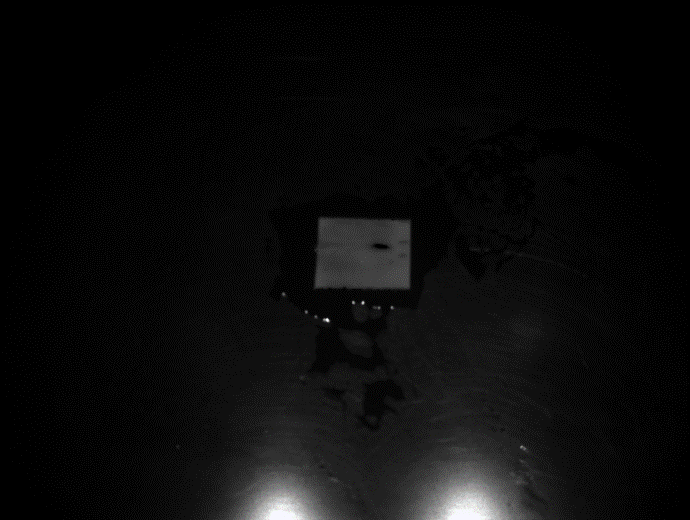


N-Cadherin


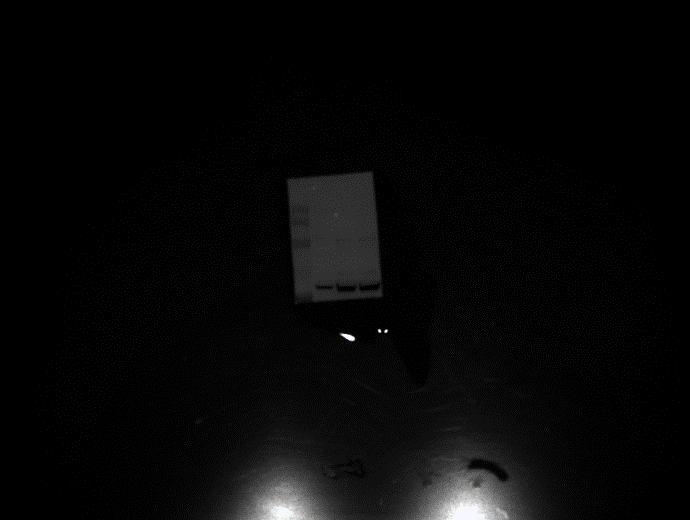


Vimentin


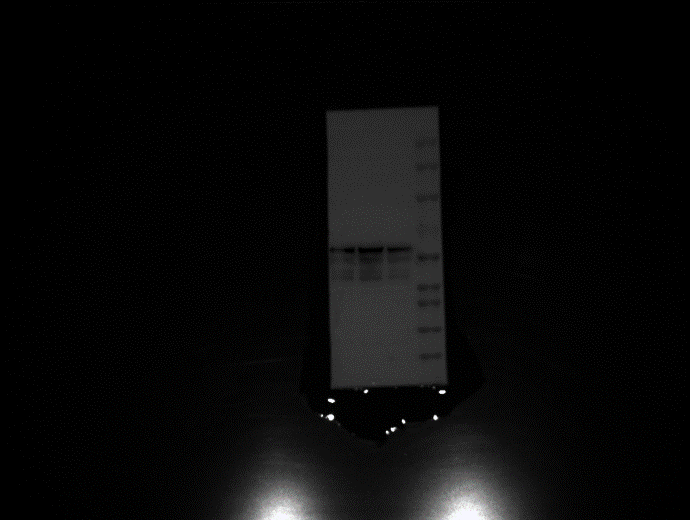


GAPDH


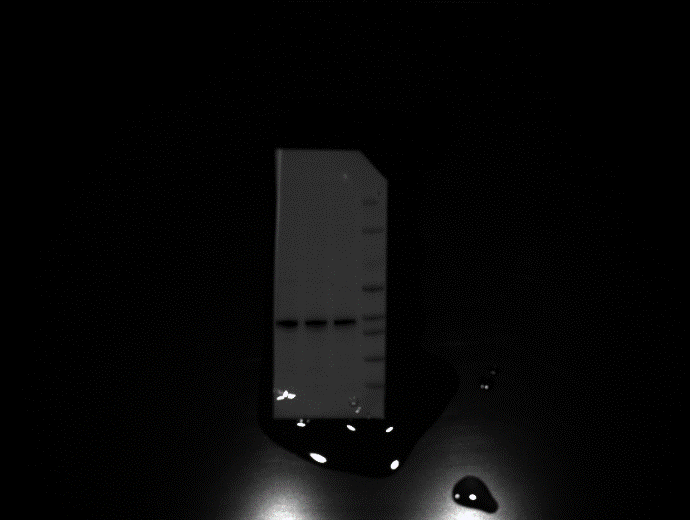


AN3CA-DCHS1 RNAi-E-cadherin


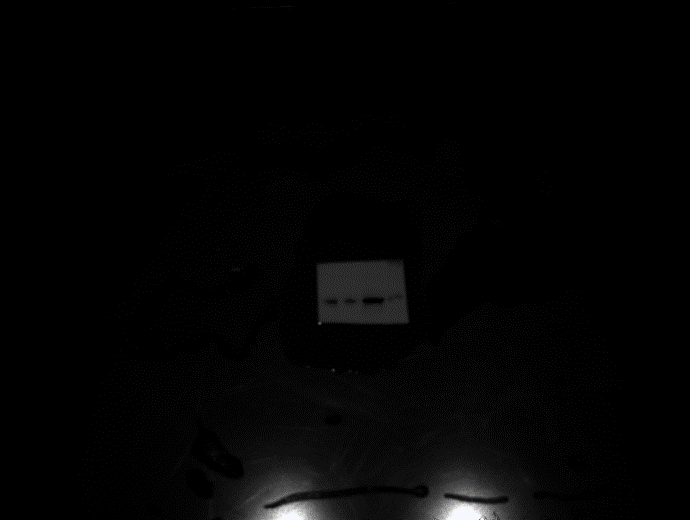


N-Cadherin


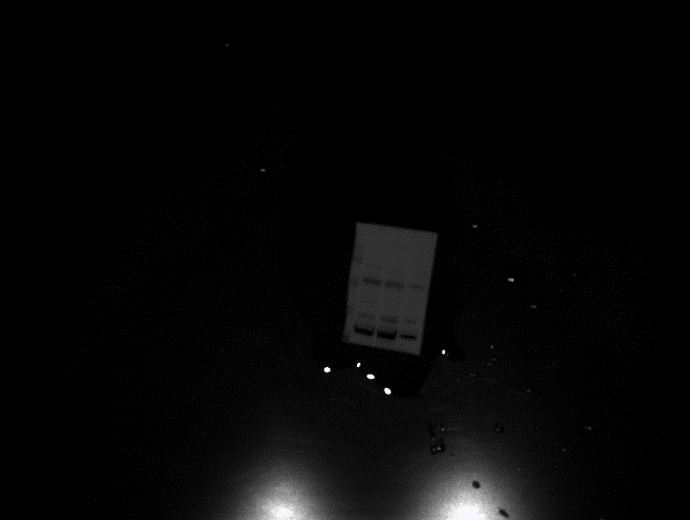


Vimentin


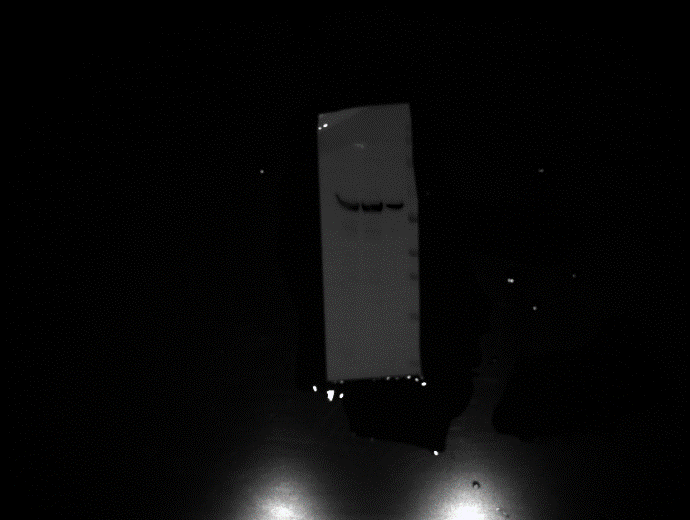


GAPDH


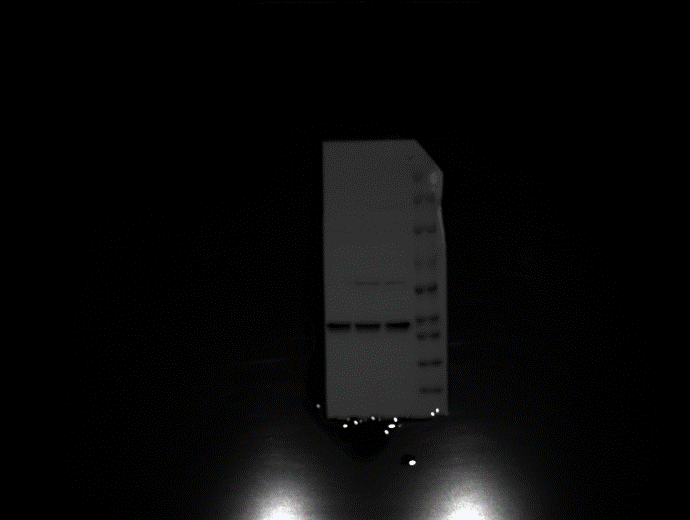


ISK-DCHS1OV-E-cadherin


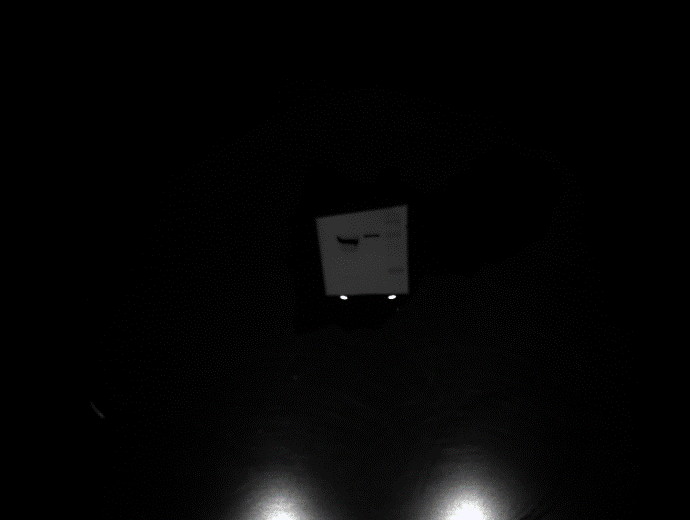


N-Cadherin


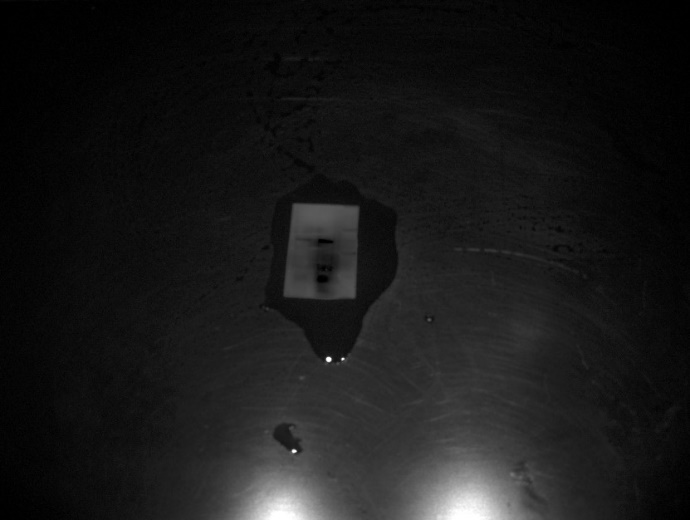


Vimentin


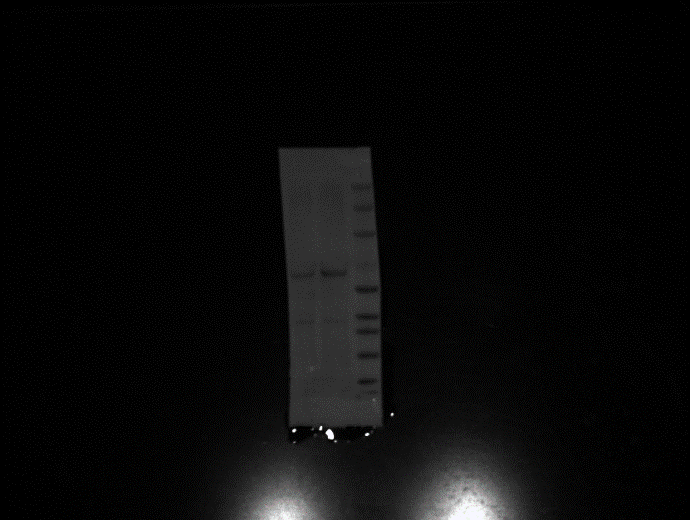


GAPDH


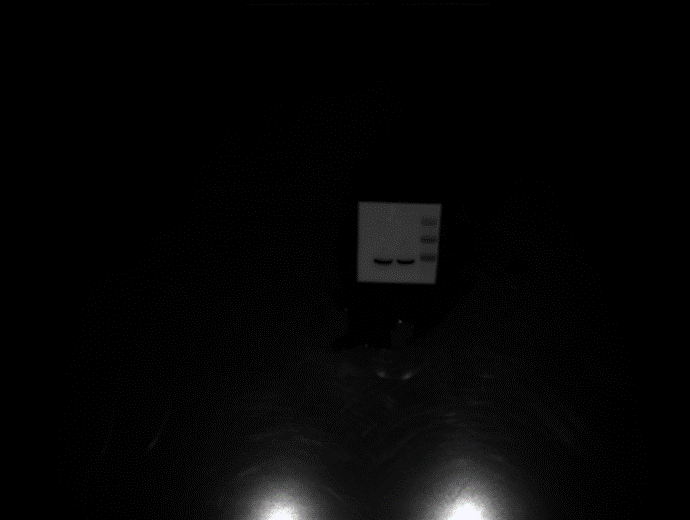


AN3CA-DCHS1OV-E-cadherin


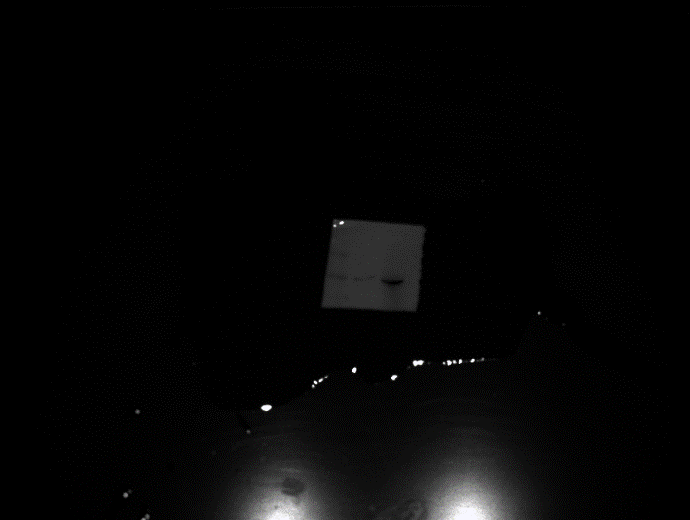


N-Cadherin


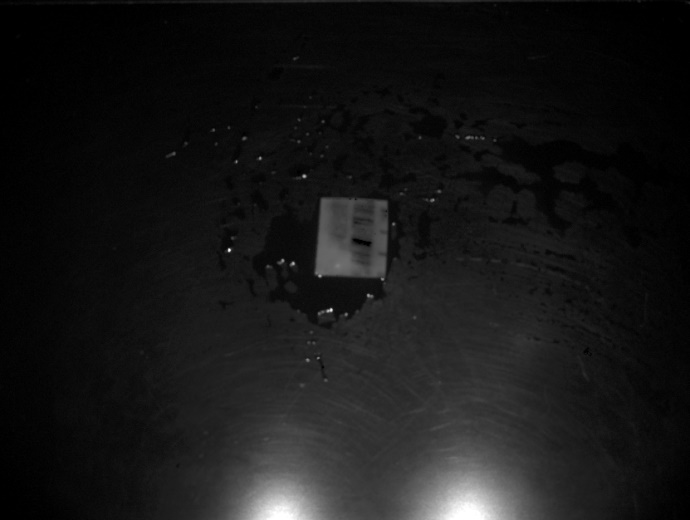


Vimentin


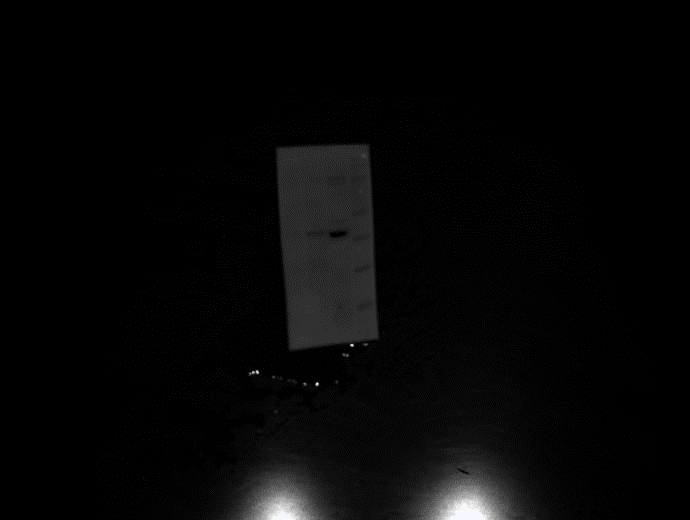


GAPDH


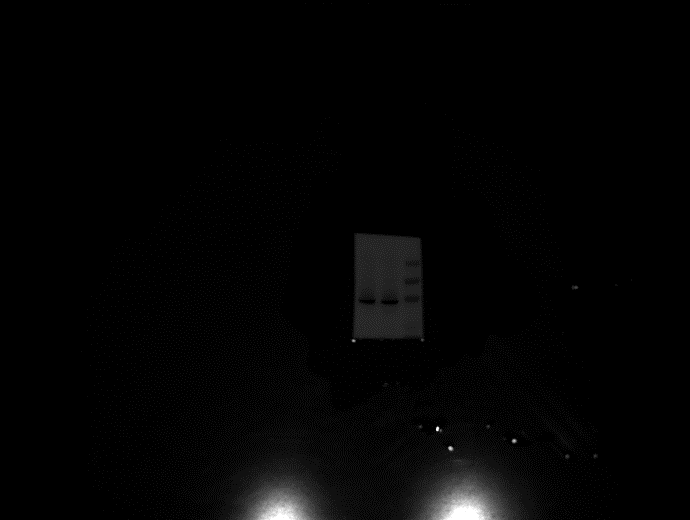

Supplement: Supplementary file 5 — Supplementary Material 5 [file 13048_2024_1478_MOESM5_ESM.docx]
